# Algorithms for incorporating prior topological information in HMMs: application to transmembrane proteins

**DOI:** 10.1186/1471-2105-7-189

**Published:** 2006-04-05

**Authors:** Pantelis G Bagos, Theodore D Liakopoulos, Stavros J Hamodrakas

**Affiliations:** 1 Department of Cell Biology and Biophysics, Faculty of Biology, University of Athens, Athens 157 01, Greece

## Abstract

**Background:**

Hidden Markov Models (HMMs) have been extensively used in computational molecular biology, for modelling protein and nucleic acid sequences. In many applications, such as transmembrane protein topology prediction, the incorporation of limited amount of information regarding the topology, arising from biochemical experiments, has been proved a very useful strategy that increased remarkably the performance of even the top-scoring methods. However, no clear and formal explanation of the algorithms that retains the probabilistic interpretation of the models has been presented so far in the literature.

**Results:**

We present here, a simple method that allows incorporation of prior topological information concerning the sequences at hand, while at the same time the HMMs retain their full probabilistic interpretation in terms of conditional probabilities. We present modifications to the standard Forward and Backward algorithms of HMMs and we also show explicitly, how reliable predictions may arise by these modifications, using all the algorithms currently available for decoding HMMs. A similar procedure may be used in the training procedure, aiming at optimizing the labels of the HMM's classes, especially in cases such as transmembrane proteins where the labels of the membrane-spanning segments are inherently misplaced. We present an application of this approach developing a method to predict the transmembrane regions of alpha-helical membrane proteins, trained on crystallographically solved data. We show that this method compares well against already established algorithms presented in the literature, and it is extremely useful in practical applications.

**Conclusion:**

The algorithms presented here, are easily implemented in any kind of a Hidden Markov Model, whereas the prediction method (HMM-TM) is freely available for academic users at , offering the most advanced decoding options currently available.

## Background

Hidden Markov Models (HMMs) are probabilistic models [1], commonly used during the last years for applications in bioinformatics [2]. These tasks include gene finding [3], multiple alignments [4] and database searches [5], prediction of signal peptides [6,7], prediction of protein secondary structure [8], prediction of transmembrane protein topology [9,10], as well as joint prediction of transmembrane helices and signal peptides [11]. Especially in the case of transmembrane proteins, HMMs have been found to perform significantly better compared to other sophisticated Machine-Learning techniques such as Neural Networks (NNs) or Support Vector Machines (SVMs). This is the case irrespective to which class of transmembrane proteins we refer to, since it has been shown that the best currently available predictors for alpha-helical membrane proteins [12,13] as well as for beta-barrel outer membrane proteins [14], are methods based on HMMs.

Experimental techniques are routinely used to partially uncover the transmembrane topology of membrane proteins (in contrast to the more expensive and difficult method of crystallography with which the detailed three-dimensional structure is elucidated). Such methods include the use of various reporter fusions [15,16], chemical labelling [17], epitope tagging [18], antibodies [19] and Cysteine scanning mutagenesis [20]. The gene fusion approach seems to be the most effective method and the reporters used, include alkaline phosphatase (PhoA), beta-galactosidase (LacZ) [21], beta-lactamase (BlaM) [22] as well as various fluorescent proteins [23]. With the development of fast and reliable methods to utilise gene fusion technology in order to determine the location of a protein's C-terminus, it has been shown that incorporating topological information into the prediction methods improves largely the performance of even the top-scoring methods, making easy to screen newly sequenced genomes [24,25]. This has been demonstrated, using experimentally derived information regarding *E. coli *[26] and *S. cerevisiae *[27] protein datasets. More recently, a global topological analysis of the *E. coli *inner membrane proteins was performed, providing reliable models for more than 600 membrane proteins [28].

Among the top-scoring HMM predictors currently available, HMMTOP [29] provides the user the option to incorporate such information. TMHMM is currently not supporting such an option in its standard web-server, and the users have to turn the TMHMMfix server [30] or to the joint prediction of transmembrane helices and signal peptides offered by Phobius [11]. Lately, Bernsel and von Heijne [31], used a similar idea that consisted of treating the occurrence (in a membrane protein) of soluble protein domains with known localisation, as experimentally determined topology. This way, they implemented a modified version of the HMM described by Viklund and Elofsson [13], in order to apply constrained predictions. However, even in this work the algorithmic details were not presented nor the prediction method became available to the public.

Moreover, no effort has been made in the literature in order to completely and accurately describe the mathematical details of the algorithms that allow the arbitrary incorporation of such prior knowledge, in a way that prevents HMMs from loosing their probabilistic interpretation. The nature of HMMs, allows some brute conditioning; for instance, setting transition probabilities from a particular state to the end state to zero will allow the fixation of the C-terminus in the desired topology. Similarly, having knowledge of the presence of a signal peptide, after removing it, one may force the HMM to consider as allowed transitions from the begin state only those with direction to the extracellular loop states of the model. Unfortunately, the probabilistic interpretation of these results will be lost. Thus, it would be useful to have a method that enables us to arbitrarily fix any part of the sequence in a specified topology, while at the same time retaining the probabilistic interpretation of the algorithms used for decoding the HMM.

In this work, we present some trivial modifications to the standard algorithms used in HMMs, namely the Forward and Backward algorithms [1,2]. These modifications are very similar with those used on training HMMs with labelled sequences [32], but here they are considered in the context of models' decoding. We also show, that the likelihoods derived when applying these modified algorithms, can be expressed as posterior probabilities of the prior experimental information given the sequences and the model parameters, thus retaining this way the probabilistic interpretation of the results. We also introduce similar trivial modifications to all known algorithms used for decoding an HMM. In particular, we present modified versions of the standard Viterbi algorithm [2], which finds the most probable path of states, of the 1-best algorithm [33], which tries to find the optimal labelling of a sequence, giving always a labelling with equal or greater probability compared to the Viterbi decoding. Similar modifications follow for the "a-posteriori" decoding method [2], which in many applications, and under certain conditions, provides better prediction [34], as well as to the newly developed Posterior-Viterbi method [35] and the Optimal Accuracy Posterior Decoding method [36], that both combine elements of Viterbi and Posterior decoding.

Finally, we present an application of these algorithms, training a HMM to predict the transmembrane segments of alpha-helical membrane proteins. The model is trained in a discriminative manner with the Conditional Maximum Likelihood (CML) criterion, using a dataset of proteins with structures known at atomic resolution, and it is shown (in cross-validation as well as in independent tests) to compare well against the top-scoring algorithms currently available. The method, HMM-TM, is freely available for academic users at , where the user may choose any of the four above mentioned algorithms for decoding, an option not currently available in any other prediction method.

## Results

In Table 1, we list the results obtained on the training set with our method (using the Optimal Accuracy Posterior Decoding), both on a self-consistency test and on a 9-fold cross-validation procedure. In the same table, for the sake of comparison, the results obtained on the same dataset are listed also, using the other available HMM and HMM-like predictors TMHMM [9], HMMTOP [29], MEMSAT [37], Phobius [11], UMDHMM^TMHP ^[38], and the newly developed S-TMHMM, PRO-TMHMM and PRODIV-TMHMM [13]. All methods are using single sequence information except from PRO-TMHMM and PRODIV-TMHMM that use evolutionary information derived from multiple alignments. The recently published and very successful method of Martelli et al [39], was not considered in the current evaluation for several reasons. Firstly, the particular method is currently not available to the public. Secondly and more importantly, this method is based in an Ensemble network combining the results of two independent HMM modules with these of a Neural Network predictor, using a dynamic programming algorithm that filters the prediction in a last step. Thus, this method even though uses HMMs it cannot be benefited directly by the currently proposed methodology. Other popular and successful methods such as PHDhtm [40,41] and TopPred [42], were also not consider in this evaluation since on the one hand our intention was to evaluate only the HMM and HMM-like predictors that are amenable to incorporate the modifications presented here, and, on the other hand, due to their lower accuracy in general [13], as also as on the particular datasets [26]. For measures of accuracy, we chose the fraction of the correctly predicted residues (Q), the correlation coefficient (C), the segments overlap (SOV), as well as the fraction of proteins with correctly predicted transmembrane segments and correctly predicted topology [43,44].

The differences in Q, C, SOV and in the number correctly predicted transmembrane segments among the methods compared here were not statistically significant (p-value>0.05 in all cases; see Materials and methods). Only an overall difference in the number of correctly predicted topologies could be detected (p-value = 0.008), which is attributable to the large discrepancies between the high-scoring methods (those using evolutionary information) and the low-scoring ones (in this case TMHMM, HMMTOP and MEMSAT). However, even this should be questionable due to the presence in the set used for training PRO-TMHMM, PRODIV-TMHMM of proteins similar to the ones we compare here.

Even though some of the proteins present in the training set were also included in the sets used for training the other predictors, HMM-TM as tested in the cross-validation test, performs better compared to methods that use single sequences. The superiority is visible (although not statistically significant) in almost all measured attributes, but it is more evident in the number of correctly predicted topologies. We have to assume that the combination of the quality of the training dataset, the CML training scheme and the label optimisation procedure that was performed using the above-mentioned algorithms, is responsible for this result, even though the training set is the one of the smallest that has ever been used for alpha-helical membrane proteins. HMM-TM, when trained and tested on the whole dataset of 72 proteins clearly outperforms also, the algorithmically simpler HMM method UMDHMM^TMHP ^that is trained on the same dataset (SOV = 0.978 and 0.933 respectively, correctly predicted topologies 94.4% and 84.7%, respectively). Compared against the methods that utilise multiple alignments, HMM-TM performs slightly worse, something already expected [13]. However the superiority of the two multiple alignment-based methods is not in the extent previously believed, considering also the presence of homologous sequences in the set used to train these methods, and the non-significant result of the Kruskal-Wallis test. The Optimal Accuracy Posterior Decoding, the Posterior decoding with the dynamic programming and the Posterior-Viterbi decoding, perform equally well, and both are superior to the 1-best and Viterbi algorithms, results which, at least for this case, are in partial agreement with those reported in [35,36].

When the performance of the methods was tested in the independent test set of 26 proteins (see Materials and methods section), similar results were obtained (Table 2). All methods (perhaps with the exception of MEMSAT) seem to perform equally well, and in all cases the performance of the topology prediction lies within the expected range.

No statistically significant differences could be found concerning Q, C, SOV and the number of correctly predicted transmembrane segments among the different methods (p > 0.05 in all cases). However, when comparing the number of correctly predicted topologies, there was a marginally significant difference with a p-value of 0.052. When excluding the three worst performing methods (Phobius, MEMSAT and PRO-TMHMM), no overall differences could be detected (p-value = 0.208). In the pairwise comparisons of HMM-TM against these three methods (without however adjusting for multiple testing), HMM-TM performs better than MEMSAT and Phobius (p-value = 0.021) and marginally better than PRO-TMHMM (p-value = 0.074). Overall, HMM-TM performs slightly better than both TMHMM and HMMTOP, and it is interesting that UMDHMM^TMHP ^performs somewhat better, even though in some cases it yields spurious predictions such as a transmembrane helix with 58 amino-acids length (1KPL:A). Interestingly, the newly developed methods (S-TMHMM, PRO-TMHMM and PRODIV-TMHMM) do not seem to perform better, despite the presence in their training set of some sequences similar to those under evaluation. Even though the independent test set consists mostly of multi-spanning (21 out of 26), and Prokaryotic proteins (22 out of the 26), its use is currently the most appropriate solution since we wanted to independently test the predictors in an, as much as larger as possible dataset, consisting of proteins having no significant similarity to the ones used for training each method. We should emphasize, that in nearly all the publications describing similar prediction methods, an independent test set was not used [9,11,13,29,36]. Only Martelli et al [39], used as an independent test set, proteins with topology determined by low-resolution biochemical experiments, and concluded that such datasets should not be used either as training or test datasets. This last argument, also applies in order to explain the fact that we were not used such proteins to further enlarge the test set making it thus more balanced.

Our prediction method was also tested on the 2 datasets containing proteins from *E. coli *[26] and *S. cerevisiae *[27](31 and 37 respectively). For reasons of brevity, the detailed results are listed in [Additional File 1]. We observe, that our method compares favourably to the other available predictors. In the *E. coli *dataset, HMM-TM predicts correctly the localization of the C-terminal part of the sequence for 29 out of the 31 proteins (93.54%), outperformed only by Phobius, which predicts correctly 30 out of the 31 proteins (96.77%). Compared against the methods using evolutionary information (PRO-TMHMM, PRODIV-TMHMM), our method performs similarly to the PRODIV-TMHMM (which predicts 29 out of the 31 proteins correctly), and better than PRO-TMHMM (28 out of 31). All the remaining methods (TMHMM, HMMTOP, MEMSAT, UMDHMM^TMHP ^and S-TMHMM) perform significantly worse, yielding from 20 to 27 correct predictions. In this dataset, the Kruskal-Wallis test yields an overall p-value of 0.0019, suggesting that there are true statistically detectable differences in the performance of the various methods. In the pairwise comparisons HMM-TM performs significantly better compared to UMDHMM^TMHP ^(p-value = 0.0054, which remains significance after adjusting for multiple comparisons) and against MEMSAT (p-value = 0.011, which does not remain significant after adjustment). The comparisons of HMM-TM against the remaining methods yielded insignificant results. Excluding from the analysis these two last methods, no overall differences could be found (p-value = 0.154). Furthermore, the two methods using evolutionary information (PRO-TMHMM, PRODIV-TMHMM) do not perform significantly better compared to the other four single-sequence methods (HMM-TM, TMHMM, HMMTOP and S-TMHMM), since the Kruskal-Wallis test yields an overall p-value of 0.314. Concerning the number of the predicted transmembrane helices, HMM-TM predicts closely to the other available top-scoring predictors. For instance, it is in agreement with the predictions obtained from TMHMM for 30 out of the 34 proteins, with those obtained from HMMTOP for 28 out of the 34, and for 29 out of the 34 proteins with those obtained from PRO-TMHMM and PRODIV-TMHMM.

In the *S. cerevisiae *dataset, top-scoring methods were found to be HMMTOP, MEMSAT, PRO-TMHMM and PRODIV-TMHMM correctly predicting the localisation for the C-terminus for 32 out of the 37 proteins (86.50%). HMM-TM, predicts correctly 30 out of the 37 proteins (81.1%), and TMHMM, S-TMHMM and UMDHMM^TMHP ^reached correct conclusions for 28 out of the 37 proteins (75.7%). Phobius in this dataset performs significantly worse, reaching an accuracy of only 70.27%. Similar observations hold also for this set, concerning the number of predicted transmembrane segments, and the general agreement of our method with the others. In this dataset however, there are not large discrepancies among the various methods resulting in an overall insignificant Kruskal-Wallis test (p-value = 0.487).

In total (summing the 2 sets), the 2 methods using evolutionary information (PRO-TMHMM and PRODIV-TMHMM), were ranked first, with 88.23% and 89.71% correct predictions respectively, followed by HMM-TM (86.76%), HMMTOP (83.82%), Phobius (82.35%), TMHMM (80.88%), MEMSAT (77.94%), S-TMHMM (76.47%) and UMDHMM^TMHP ^(70.59%). However, these differences showed no overall statisticall significance (p-value = 0.086).

**Figure 1:**
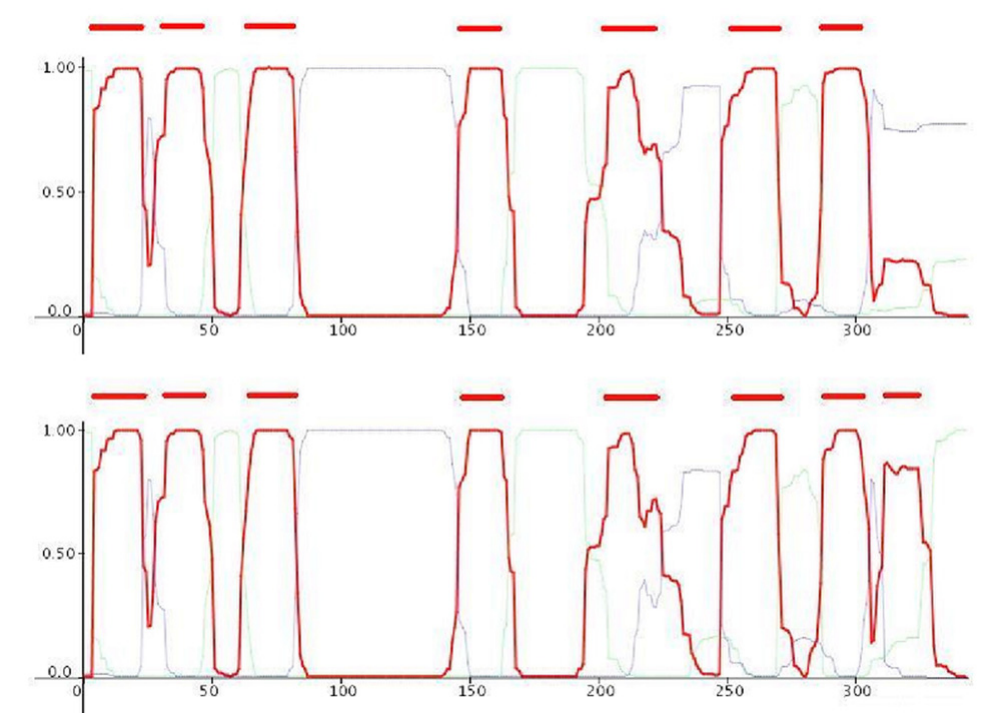
**Posterior probability plots and predicted transmembrane segments for a protein whose localisation of the C-terminal was missed by HMM-TM (YDGG_ECOLI)**. In the upper graph we can see the unconstrained prediction. In the lower part, we can see the conditional prediction, after incorporating the information concerning the experimentally verified localisation of the C-terminus. The red bars indicate the predicted transmembrane segments, and we observe that these change also, coming in agreement with the other predictors.

Even though, the methods using evolutionary information perform slightly (in some datasets) better than HMM-TM, their superiority is not validated statistically here and more importantly, they do not offer the option to fix the topology of various sequence parts. Only TMHMM, Phobius and HMMTOP offer such options, and compared to them, HMM-TM seems to perform constantly better in all the tests performed. Interestingly, when the experimentally determined topology of the C-terminus is incorporated into the predictions for the two proteins on which the method failed (YDGG_ECOLI, ZITB_ECOLI), the predicted number of transmembrane segments changes (from 7 to 8 and from 7 to 6, respectively) and shows a remarkable agreement with those predicted by the above-mentioned methods (figure 1). Using the same reasoning for the proteins missed by HMMTOP and TMHMM, we can reach similar conclusions. In the independent dataset of 26 proteins, if we fix the location of the C-terminus to its observed topology, all of the methods that are capable of incorporating such information (HMM-TM, TMHMM, HMMTOP and Phobius), increase both the number of correctly predicted transmembrane segments and the number of correctly predicted topologies. In HMM-TM the number of correctly predicted transmembrane segments is increased from 21 to 22, as well as the number of correctly predicted topologies (from 21 to 22). Similarly, in TMHMM number of correctly predicted topologies becomes 19 (from 17), while the number of correctly predicted transmembrane segments remains 19; in HMMTOP we observe only a slight increase of the correctly predicted topologies (19 from 18) and finally for Phobius we observe an increase both in the correctly predicted topologies becomes (16 from 13) and in the correctly predicted transmembrane segments (16 from 15). Similar results were presented initially in the work of Mellen, Krogh and von Heijne [24], but here we could not validate them statistically due to the small sample size. Thus, this observation should be further tested in a larger independent dataset of proteins with experimentally determined topology. We have to mention though, that there is a clear gain in correctly specifying the topology using such algorithms, and in future works it might be tempting to construct a predictor that uses both evolutionary derived information and the option to fix various sequence parts in the desired location.

**Figure 2:**
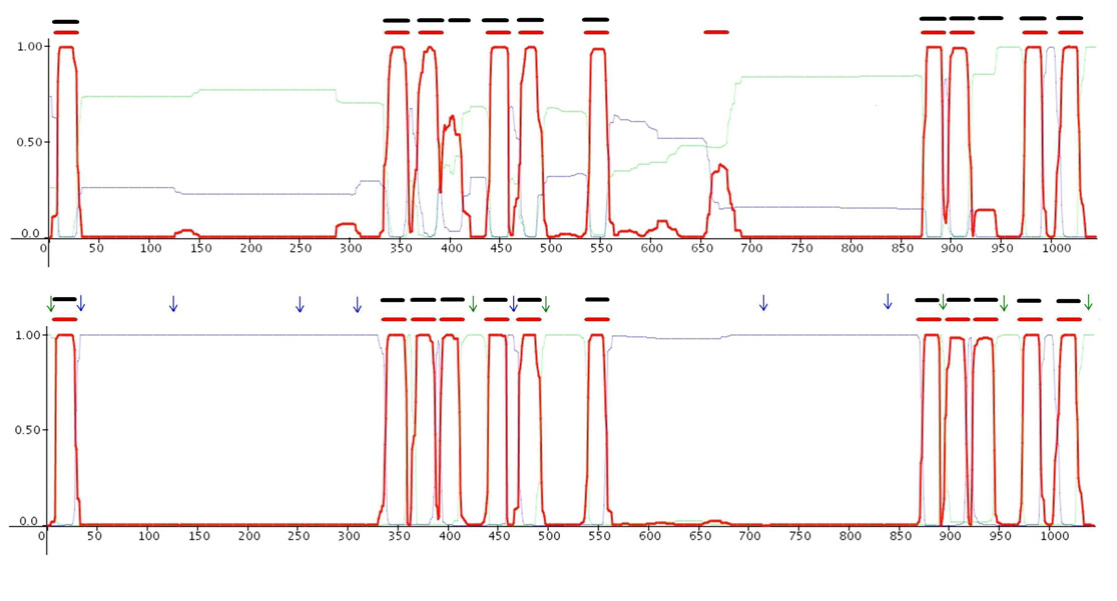
**Posterior probability plots and predicted transmembrane segments for the multidrug efflux transporter AcrB, a protein with known 3-dimensional structure **(PDB code: 1IWG). In the upper graph we can see the unconstrained prediction. The red bars indicate the predicted transmembrane segments whereas the black bars, the observed segments. There are two missed transmembrane helices and a falsely predicted one. In the lower part, we can see the constrained prediction, after incorporating the experimental information derived from cysteine-scanning mutagenesis experiments [46]. Green arrows indicate the experimentally verified localisation of a residue in the cytoplasm, whereas blue ones indicate the experimentally verified localisation to the extracellular (periplasmic) space. We observe a remarkable agreement of the constrained prediction with the known structure.

The usefulness and the practical applicability of the prediction method described here, combined with the option of fixing the topology of various segments along the sequence incorporating this way the experimental information during the decoding phase, could be demonstrated in the case of the multidrug efflux transporter AcrB of *E. coli*. The structure has been resolved crystallographically [45], and it has been shown that the protein contains 12 transmembrane helices (AcrB is included in the blind test set of 26 proteins used in this study). All the prediction methods used here (with the exception of S-TMHMM, PRO-TMHMM and PRODIV-TMHMM, which in their training set used a close homologue, MexB) failed to accurately predict the full topology of the protein. As we can see (Figure 2, upper part), HMM-TM misses 2 transmembrane segments, while falsely predicts an additional segment that does not exist. However, using the results obtained from cysteine-scanning mutagenesis [46], and incorporating them in the predictions obtained with HMM-TM (Figure 2, lower part), results in a highly reliable prediction that is in excellent agreement with the obtained crystallographically solved structure.

Lastly, even though this was not a primary intention of our study, we evaluated the discrimination capabilities of the methods on the set of 645 globular proteins with known three-dimensional structures, a set used initially for the same purpose in the evaluation of TMHMM [9]. The results were very conflicting reflecting the differences in the training strategies of the various predictors as well as the different primary intentions of their developers. Thus, when the total number of proteins predicted to be non transmembrane was evaluated, methods that were designed to be applied only on transmembrane proteins such as HMM-TM, HMMTOP, UMDHMM^TMHP^, MEMSAT and PRODIV-TMHMM perform poorly, predicting falsely 15.5%, 8.37%, 24.18%, 99.84% and 76.89% of the globular proteins to possess at least one transmembrane segment, respectively. From the other hand, methods that were trained initially in order to discriminate globular proteins from transmembrane ones, such as TMHMM, Phobius, S-TMHMM and PRO-TMHMM, perform considerably better, predicting respectively only 0.62%, 1.1%, 1.7% and 0.62% of the globular proteins to be transmembrane. When we considered as a discrimination criterion the total number of aminoacids predicted in transmebrane segments, and taking as a cut-off length the value of 19 aminoacids, HMM-TM predicts falsely 5.73% of the globular proteins, TMHMM 0.46%, HMMTOP 4.96%, Phobius 0.9%, UMDHMM^TMHP ^11.32%, MEMSAT 14.42%, S-TMHMM 1.7%, PRO-TMHMM 0.62% and PRODIV-TMHMM 76.89%. The differences in both cases are highly significant (p-values<0.001), and it is evident that PRODIV-TMHMM is highly unreliable for discrimination purposes, a fact noticed already in [13], followed by MEMSAT and UMDHMM^TMHP^, whereas HMM-TM and HMMTOP form a group of intermediate reliability just before the highly reliable for this task methods, TMHMM, Phobius, S-TMHMM and PRO-TMHMM.

## Discussion

The primary intention of this work was to introduce the modifications to the standard algorithms that will allow incorporation of prior structural or topological information during the decoding procedure; however, the prediction method presented is quite accurate. One may argue, that we should have trained a prediction method that uses evolutionary information in order to achieve the best possible results [13]. However, this was not possible currently for technical reasons. Furthermore, as we already stated, the primary intention of the particular work was to present the algorithmic details, and not to develop a prediction method. Thus, the algorithmic modifications could now be easily applied in any prediction method based on a HMM.

From the reported results, it is obvious, that HMM-TM performs comparably if not better than the already established top-scoring HMM methods using single sequences and compares well against the methods using multiple alignments. As a matter of fact, using a rigorous statistical methodology, we found that HMM-TM is not outperformed significantly in any of the tests presented here (except for the test discrimination capabilities). On the contrary, clearly outperforms some of the available methods in some of the performed tests. These conclusions, are valid also for the per-residue and per-segment measures reported in the set of 72 proteins, as well as for the blind test on newly solved three-dimensional structures and on proteins with experimentally verified location of the C-terminus. However, there are cases in which a joint prediction could give better results than each one of the individual methods. From the detailed results listed in [Additional File 1], we observe that for only 2 out of the 68 proteins used (YNT4_YEAST, Q03193) all of the available algorithms fail to predict correctly the localisation of the C-terminal. This yields another potential use for HMM-TM besides acting as a standalone predictor: it could be very useful as a part of a consensus prediction method. Such consensus predictions, have been proven to significantly improve the predictive performance compared to individual methods, either referring to alpha-helical [47-49], beta-barrel membrane proteins [14] or to general secondary structure prediction methods [50]. For developing a successful consensus prediction method, it is necessary to have high-scoring individual methods, producing independent results, which indeed is the case here.

We should emphasize also at this point, that the algorithmic modifications that we introduce, by no way confer bias to the prediction method. Thus, when the decoding (prediction) is applied in an unconstrained manner, the results obtained are not by any means are affected by the existence of the modifications since they do not used at all. However, when experimentally derived information emerges, the algorithmic modifications force the predictions to be in agreement with this prior information. Thus, in such cases it is reasonable to observe better predictions obtained by such an algorithm (that uses the prior information) compared to another algorithm (even a superior one) that does not use this information. Ideally, in future works these modifications should be applied to a prediction method that uses evolutionary information in order to obtain the best possible results. Furthermore, the extent to which the prior knowledge affects the rate of the correct predictions should be evaluated in a larger dataset.

The algorithmic modifications presented in this paper (see Materials and methods), are for the first time introduced in such detail, allowing one to implement them in a straightforward manner. Although these modifications appear trivial, there is clearly a need to be described in such a detail in order to further clarify some obscure points in the literature. All the relevant works addressed such an issue, in the past were either published as a short applications note in Computational Biology journals, or as regular papers in Molecular Biology journals, and in both cases they did not spend more than a sentence or two for describing the algorithms (with in some cases contradicting notations). For instance, in the first published work that mentions a constraint prediction [29], the authors state (emphasis added from us): "This segment information is incorporated into the *Baum-Welch algorithm by a conditional probability*." In a later work [24], the authors simply state that: "The basic TMHMM algorithm allows one to fix the class-assignment for any position in the sequence *by setting the probability for a position to belong to a certain class to 1.0 a *priori." Finally, in a recently published work [31], Bernsel and von Heijne state that: "The IN/OUT-fixation of a certain residue is *achieved by setting the corresponding state probability in the HMM equal to 1.0, ..."*. Clearly, these statements do not constitute complete and thoroughly described methodology that it is easily applicable from someone willing to incorporate such options to a prediction method.

Furthermore, here for the first time we apply algorithms for constrained predictions applicable to all the currently available decoding methods, and in all cases preserving the probabilistic nature of the HMM. It is clear though, that these modifications can also be used in any decoding algorithm may appear in the future, or in any HMM trained with various schemes (ML or CML), as well as in methods using evolutionary information or not. Lastly, we should also point that in this work we considered only incorporating in the prediction the experimentally verified topology of various sequence segments. Other works have been presented, dealing for instance with the incorporation of prior physicochemical knowledge concerning the propensity of a helix to be transmembrane or not [51]. Clearly, such approaches even though useful in practical applications, are irrelevant to the currently proposed methodology and should be considered separately.

## Conclusion

We have presented here, modified algorithms for incorporating prior topological information into HMMs. These algorithms constitute trivial modifications to the well known Forward and Backward algorithms involved in the probability calculations on HMMs, as well as to the already established algorithms for decoding such models (Viterbi, 1-best, and the variants of posterior decoding). We presented these algorithms without introducing further computational complexity, while at the same time retaining the probabilistic interpretation of the results. These algorithms may also be useful in other applications of HMMs, besides the transmembrane protein topology prediction, since they could be applied in any circular HMM, irrespective of the training procedure used. We have shown that these algorithms could be used also with more complex labelling schemes as well as with HMMs using both discrete and continuous emission probabilities. The same modifications may also be applied in optimising the discriminative capability of the models, especially in cases of misplaced labelling arising from inherently mislabelled data. We have presented an application in the prediction of transmembrane segments of alpha-helical membrane proteins, and we developed a method that compares well against, if not better than, the already available top-scoring methods for the same task either using single sequences or multiple alignments. We also have to note, that the method presented here undoubtedly seems to perform better compared to the other HMM predictors that allow the incorporation of experimentally derived information. We also confirmed the results of previous studies, indicating that incorporation of prior topological knowledge will further improve the performance of predictive algorithms, and provided evidence that using a consensus of the top-scoring methods, the predictive performance increases. Consensus of individual methods has been proven a useful strategy for obtaining better predictions, and thus, it could be benefited from including even more high-scoring individual methods. Finally, we set up a web-server, freely available to academic users, where the above-mentioned predictive method can be found (), offering the most advanced decoding options currently available. The method developed here might be useful to experimentalists who require reliable predictions, in the light of experimentally derived information.

## Methods

### Hidden Markov models

Two states *k, l *of a Hidden Markov model are connected by means of the transition probabilities *α*_*kl*_. Assuming a protein sequence **x **of length *L *denoted as:

**x **= *x_1_*, *x_2_*, ...,*x_L_*,     (1)

where the *x*_*i*_'s are the 20 amino acids, we usually denote the "path" (i.e. the sequence of states) ending up to a particular position of the amino acid sequence (the sequence of symbols), by *π*. Each state *k *is associated with an emission probability *e_k_(x_i_)*, which is the probability of a particular symbol *x*_*i *_to be emitted by that state. Formally, we also need two other special states, named *begin (B) *and *end state (E)*, which are essential for starting and ending the process, respectively. These states however, do not emit any symbol. The total probability of a sequence given the model, is calculated by summing over all possible paths using the Forward or the Backward algorithm:

P(x|θ)=∑πP(x,π|θ)=∑πaBπ1∏i=1eπi(xi)aπiπi+1     (2)
 MathType@MTEF@5@5@+=feaafiart1ev1aaatCvAUfKttLearuWrP9MDH5MBPbIqV92AaeXatLxBI9gBaebbnrfifHhDYfgasaacH8akY=wiFfYdH8Gipec8Eeeu0xXdbba9frFj0=OqFfea0dXdd9vqai=hGuQ8kuc9pgc9s8qqaq=dirpe0xb9q8qiLsFr0=vr0=vr0dc8meaabaqaciaacaGaaeqabaqabeGadaaakeaacqWGqbaudaqadaqaaGqabiab=Hha4jabcYha8HGaciab+H7aXbGaayjkaiaawMcaaiabg2da9maaqafabaGaemiuaa1aaeWaaeaacqWF4baEcqGGSaalcqGFapaCcqGG8baFcqGF4oqCaiaawIcacaGLPaaaaSqaaiab+b8aWbqab0GaeyyeIuoakiabg2da9maaqafabaGaemyyae2aaSbaaSqaaiabdkeacjab+b8aWnaaBaaameaacqaIXaqmaeqaaaWcbeaaaeaacqGFapaCaeqaniabggHiLdGcdaqeqbqaaiabdwgaLnaaBaaaleaacqGFapaCcqWGPbqAaeqaaOWaaeWaaeaacqWG4baEdaWgaaWcbaGaemyAaKgabeaaaOGaayjkaiaawMcaaiabdggaHnaaBaaaleaacqGFapaCdaWgaaadbaGaemyAaKgabeaaliab+b8aWnaaBaaameaacqWGPbqAcqGHRaWkcqaIXaqmaeqaaaWcbeaakiaaxMaacaWLjaWaaeWaaeaacqaIYaGmaiaawIcacaGLPaaaaSqaaiabdMgaPjabg2da9iabigdaXaqab0Gaey4dIunaaaa@696A@

The generalization from one to many sequences is trivial, and we will consider only one training sequence **x **in the following. When using labelled sequences for training, each amino acid sequence **x **is accompanied by a sequence of labels **y **for each position *i *in the sequence:

**y **= *y_1_*, *y_2_*, ...,*y_L _*    (3)

Krogh [32] proposed a simple modified version of the forward and backward algorithms, in order to incorporate information from labelled data. The likelihood to be maximized in such situations is the joint probability of the sequences and the labelling given the model:

P(x,y|θ)=∑πP(x,y,π|θ)=∑π∈ΠyP(x,π|θ)     (4)
 MathType@MTEF@5@5@+=feaafiart1ev1aaatCvAUfKttLearuWrP9MDH5MBPbIqV92AaeXatLxBI9gBaebbnrfifHhDYfgasaacH8akY=wiFfYdH8Gipec8Eeeu0xXdbba9frFj0=OqFfea0dXdd9vqai=hGuQ8kuc9pgc9s8qqaq=dirpe0xb9q8qiLsFr0=vr0=vr0dc8meaabaqaciaacaGaaeqabaqabeGadaaakeaacqWGqbaudaqadaqaaGqabiab=Hha4jab=XcaSiab=Lha5jabcYha8HGaciab+H7aXbGaayjkaiaawMcaaiabg2da9maaqafabaGaemiuaa1aaeWaaeaacqWF4baEcqWFSaalcqWF5bqEcqGGSaalcqGFapaCcqGG8baFcqGF4oqCaiaawIcacaGLPaaaaSqaaiab+b8aWbqab0GaeyyeIuoakiabg2da9maaqafabaGaemiuaa1aaeWaaeaacqWF4baEcqGGSaalcqGFapaCcqGG8baFcqGF4oqCaiaawIcacaGLPaaacaWLjaGaaCzcamaabmaabaGaeGinaqdacaGLOaGaayzkaaaaleaacqGFapaCcqGHiiIZcqqHGoaudaWgaaadbaGaemyEaKhabeaaaSqab0GaeyyeIuoaaaa@5EDF@

This way, the summation has to be done only over those paths Π_*y *_that are in agreement with the labels **y**. Consequently, one has to declare a new probability distribution, the probability *λ*_*k*_*(c) *of a state *k *having a label *c*. In most of the biological applications, this probability is just a delta-function, since a particular state is not allowed to match more than one label.

λk(c){1,if k∈σc0,if k∉σc     (5)
 MathType@MTEF@5@5@+=feaafiart1ev1aaatCvAUfKttLearuWrP9MDH5MBPbIqV92AaeXatLxBI9gBaebbnrfifHhDYfgasaacH8akY=wiFfYdH8Gipec8Eeeu0xXdbba9frFj0=OqFfea0dXdd9vqai=hGuQ8kuc9pgc9s8qqaq=dirpe0xb9q8qiLsFr0=vr0=vr0dc8meaabaqaciaacaGaaeqabaqabeGadaaakeaaiiGacqWF7oaBdaWgaaWcbaGaem4AaSgabeaakmaabmaabaGaem4yamgacaGLOaGaayzkaaWaaiqaaeaafaqabeGabaaabaGaeGymaeJaeiilaWIaeeyAaKMaeeOzayMaeeiiaaIaem4AaSMaeyicI4Sae83Wdm3aaSbaaSqaaiabdogaJbqabaaakeaacqaIWaamcqGGSaalcqqGPbqAcqqGMbGzcqqGGaaicqWGRbWAcqGHjiYZcqWFdpWCdaWgaaWcbaGaem4yamgabeaaaaGccaWLjaGaaCzcamaabmaabaGaeGynaudacaGLOaGaayzkaaaacaGL7baaaaa@4E92@

where *σ*_*c *_is the set of states sharing the same label (c). Labelled sequences are used in the training phase (either by Maximum Likelihood or by Conditional Maximum Likelihood), since in the decoding phase in which we do not know the path, the likelihood that is been calculated is of the form of equation (2). An HMM could also be trained using unlabelled sequences. However, having a complex model comprising of a lot of states, even in the decoding phase, we eventually have to cluster the states in "classes" that each one represents some biological meaningful entity. For instance, in the case of transmembrane proteins, these labels (classes) would probably be membrane (M), intracellular (I) and extracellular or outer (O), irrespective of which method the model was trained with. Thus, in the following, we will use the concept of labels in the decoding phase, no matter what training technique has been followed.

The algorithms that we present here are also very useful in the training phase using labelled sequences, under certain circumstances. For instance, in transmembrane protein topology prediction, even if the observed labels come from crystallographically solved structures, we cannot locate precisely the boundaries of the lipid bilayer. Thus, these inherently misplaced labels may bias the training, towards poor discriminative capability. In some of the most successful models for predicting the membrane-spanning alpha helices [9,11,13], an optimisation procedure was used, according to which the model was trained initially, the labels were partially disregarded around the ends of the transmembrane helices, and predictions conditional on the remaining labels were performed, in order to re-label the data until the final training procedure. The authors of these methods did not explicitly provide details on the algorithms they used, but the most obvious way for such a procedure to be performed is by using some algorithmic modifications such as those presented here.

### Forward and Backward algorithms

The standard algorithm employed in the likelihood calculations in HMMs, is the Forward algorithm. It is a dynamic programming algorithm that sums iteratively the probabilities of all the possible paths through the sequence. The algorithm as presented in [2] is:

#### Forward algorithm

∀k≠B,i=0:fB(0)=1,fk(0)=0,∀1≤i≤L:fl(i)=el(xi)∑kfk(i−1)akl     (6)P(x|θ)=∑kfk(L)akE
 MathType@MTEF@5@5@+=feaafiart1ev1aaatCvAUfKttLearuWrP9MDH5MBPbIqV92AaeXatLxBI9gBaebbnrfifHhDYfgasaacH8akY=wiFfYdH8Gipec8Eeeu0xXdbba9frFj0=OqFfea0dXdd9vqai=hGuQ8kuc9pgc9s8qqaq=dirpe0xb9q8qiLsFr0=vr0=vr0dc8meaabaqaciaacaGaaeqabaqabeGadaaakqaabeqaaiabgcGiIiabdUgaRjabgcMi5kabdkeacjabcYcaSiabdMgaPjabg2da9iabicdaWiabcQda6iabdAgaMnaaBaaaleaacqWGcbGqaeqaaOWaaeWaaeaacqaIWaamaiaawIcacaGLPaaacqGH9aqpcqaIXaqmcqGGSaalcqWGMbGzdaWgaaWcbaGaem4AaSgabeaakmaabmaabaGaeGimaadacaGLOaGaayzkaaGaeyypa0JaeGimaaJaeiilaWcabaGaeyiaIiIaeGymaeJaeyizImQaemyAaKMaeyizImQaemitaWKaeiOoaOJaemOzay2aaSbaaSqaaiabdYgaSbqabaGcdaqadaqaaiabdMgaPbGaayjkaiaawMcaaiabg2da9iabdwgaLnaaBaaaleaacqWGSbaBaeqaaOWaaeWaaeaacqWG4baEdaWgaaWcbaGaemyAaKgabeaaaOGaayjkaiaawMcaamaaqafabaGaemOzay2aaSbaaSqaaiabdUgaRbqabaGcdaqadaqaaiabdMgaPjabgkHiTiabigdaXaGaayjkaiaawMcaaiabdggaHnaaBaaaleaacqWGRbWAcqWGSbaBaeqaaaqaaiabdUgaRbqab0GaeyyeIuoakiaaxMaacaWLjaWaaeWaaeaacqaI2aGnaiaawIcacaGLPaaaaeaacqWGqbaudaqadaqaaGqabiab=Hha4jabcYha8HGaciab+H7aXbGaayjkaiaawMcaaiabg2da9maaqafabaGaemOzay2aaSbaaSqaaiabdUgaRbqabaGcdaqadaqaaiabdYeambGaayjkaiaawMcaaiabdggaHnaaBaaaleaacqWGRbWAcqWGfbqraeqaaaqaaiabdUgaRbqab0GaeyyeIuoaaaaa@8682@

We will define the concept of the *Information*, **ω **that consists of 1≤*r*≤*L *residues of the sequence **x**, of which we know the experimentally determined topology, and thus the (*a priori*) labelling *ω*_*i*_:

**ω **= *ω_1_*, *ω_2_*, ...,*ω*_*r *_    (7)

According to this terminology, the set of residues with *a priori *known labels *ω*^*r*^, is a subset of the set *I *= {1,2, ...,*L*} defined by the residues of the sequence. It is obvious that the labels *ω*_*i*_, should belong to the same set of labels defined in the model architecture. Finally, we have to declare a delta function with values given by:

dk(i)={0,if λk(ωi)≠0 and i∈ωr1,otherwise     (8)
 MathType@MTEF@5@5@+=feaafiart1ev1aaatCvAUfKttLearuWrP9MDH5MBPbIqV92AaeXatLxBI9gBaebbnrfifHhDYfgasaacH8akY=wiFfYdH8Gipec8Eeeu0xXdbba9frFj0=OqFfea0dXdd9vqai=hGuQ8kuc9pgc9s8qqaq=dirpe0xb9q8qiLsFr0=vr0=vr0dc8meaabaqaciaacaGaaeqabaqabeGadaaakeaacqWGKbazdaWgaaWcbaGaem4AaSgabeaakmaabmaabaGaemyAaKgacaGLOaGaayzkaaGaeyypa0ZaaiqaaeaafaqaaeGabaaabaGaeGimaaJaeiilaWIaeeyAaKMaeeOzayMaeeiiaaccciGae83UdW2aaSbaaSqaaiabdUgaRbqabaGcdaqadaqaaiab=L8a3naaBaaaleaacqWGPbqAaeqaaaGccaGLOaGaayzkaaGaeyiyIKRaeeimaaJaeeiiaaIaeeyyaeMaeeOBa4MaeeizaqMaeeiiaaIaemyAaKMaeyicI4Sae8xYdC3aaWbaaSqabeaacqWGYbGCaaaakeaacqaIXaqmcqGGSaalcqqGVbWBcqqG0baDcqqGObaAcqqGLbqzcqqGYbGCcqqG3bWDcqqGPbqAcqqGZbWCcqqGLbqzaaGaaCzcaiaaxMaadaqadaqaaiabiIda4aGaayjkaiaawMcaaaGaay5Eaaaaaa@62A5@

**Figure 3:**
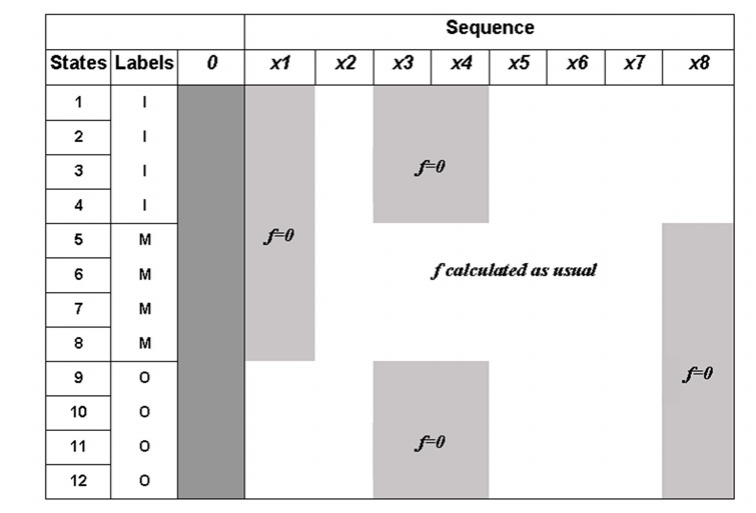
**A representation of the matrix produced by the forward algorithm modified to incorporate some prior information**. We have a (hypothetical) model, which consists of 12 states, with 3 labels I, M, O corresponding respectively to states modelling the intracellular, transmembrane and extracellular parts of the sequence. The likelihood of sequence **x **(8 residues), is calculated incorporating the prior information that residues 3 and 4 are transmembrane, residue 1 is extracellular and residue 8 is intracellular.

The trivial modification to the Forward algorithm consists simply of setting the forward variable *f *equal to zero, for each position *i *and state *k *that is not in agreement with the prior experimental information (Figure 3). This is conceptually similar with the training with labelled sequences, where we allow only those paths Π_*y *_that are in agreement with the labelling **y**, to contribute to the total likelihood. Here, we allow only the paths Π_*ω *_that are in agreement with the prior information **ω**. Thus, the modified Forward algorithm is:

#### Modified Forward algorithm

i=0:fBω(0)=1,fkω(0)=0,∀k≠B∀1≤i≤L:flω(i)=dl(i)el(xi)∑kfkω(i−1)akl     (9)P(x,ω|θ)=∑kfkω(L)akE
 MathType@MTEF@5@5@+=feaafiart1ev1aaatCvAUfKttLearuWrP9MDH5MBPbIqV92AaeXatLxBI9gBaebbnrfifHhDYfgasaacH8akY=wiFfYdH8Gipec8Eeeu0xXdbba9frFj0=OqFfea0dXdd9vqai=hGuQ8kuc9pgc9s8qqaq=dirpe0xb9q8qiLsFr0=vr0=vr0dc8meaabaqaciaacaGaaeqabaqabeGadaaakqaabeqaaiabdMgaPjabg2da9iabicdaWiabcQda6iabdAgaMnaaDaaaleaacqWGcbGqaeaaiiGacqWFjpWDaaGcdaqadaqaaiabicdaWaGaayjkaiaawMcaaiabg2da9iabigdaXiabcYcaSiabdAgaMnaaDaaaleaacqWGRbWAaeaacqWFjpWDaaGcdaqadaqaaiabicdaWaGaayjkaiaawMcaaiabg2da9iabicdaWiabcYcaSiabgcGiIiabdUgaRjabgcMi5kabdkeacbqaaiabgcGiIiabigdaXiabgsMiJkabdMgaPjabgsMiJkabdYeamjabcQda6iabdAgaMnaaDaaaleaacqWGSbaBaeaacqWFjpWDaaGcdaqadaqaaiabdMgaPbGaayjkaiaawMcaaiabg2da9iabdsgaKnaaBaaaleaacqWGSbaBaeqaaOWaaeWaaeaacqWGPbqAaiaawIcacaGLPaaacqWGLbqzdaWgaaWcbaGaemiBaWgabeaakmaabmaabaGaemiEaG3aaSbaaSqaaiabdMgaPbqabaaakiaawIcacaGLPaaadaaeqbqaaiabdAgaMnaaDaaaleaacqWGRbWAaeaacqWFjpWDaaGcdaqadaqaaiabdMgaPjabgkHiTiabigdaXaGaayjkaiaawMcaaiabdggaHnaaBaaaleaacqWGRbWAcqWGSbaBaeqaaaqaaiabdUgaRbqab0GaeyyeIuoakiaaxMaacaWLjaWaaeWaaeaacqaI5aqoaiaawIcacaGLPaaaaeaacqWGqbaudaqadaqaaGqabiab+Hha4jabcYcaSGGabiab9L8a3jabcYha8jab=H7aXbGaayjkaiaawMcaaiabg2da9maaqafabaGaemOzay2aa0baaSqaaiabdUgaRbqaaiab=L8a3baakmaabmaabaGaemitaWeacaGLOaGaayzkaaGaemyyae2aaSbaaSqaaiabdUgaRjabdweafbqabaaabaGaem4AaSgabeqdcqGHris5aaaaaa@9712@

We have to note here, that all algorithms presented hereinafter will fail to produce results when the prior information that is provided is inconsistent with the model at hand. For example, in some models we may allow only some states to have non-zero transitions from the begin state. This is the case of beta-barrel outer membrane proteins of which we know that the sequence must start by an intracellular loop [10]. If we force the model to fix the N-terminal location into extracellular space, the algorithms will produce no valid results. Furthermore, if the prior information consists of various segments, these should also be self-consistent and in accordance with the model. For instance, in transmembrane protein topology prediction, we cannot fix the position of the residue *i *to be intracellular and that of the *i*+*1*, to be extracellular, because this will also violate the model's assumptions.

The counterpart of the Forward algorithm is the Backward algorithm, which is presented below:

#### Backward algorithm

∀k,i=L:bk(L)=akE∀1≤i<L:bk(i)=∑laklel(xi+1)bl(i+1)     (10)P(x|θ)=∑laBlel(x1)bl(1)
 MathType@MTEF@5@5@+=feaafiart1ev1aaatCvAUfKttLearuWrP9MDH5MBPbIqV92AaeXatLxBI9gBaebbnrfifHhDYfgasaacH8akY=wiFfYdH8Gipec8Eeeu0xXdbba9frFj0=OqFfea0dXdd9vqai=hGuQ8kuc9pgc9s8qqaq=dirpe0xb9q8qiLsFr0=vr0=vr0dc8meaabaqaciaacaGaaeqabaqabeGadaaakqaabeqaaiabgcGiIiabdUgaRjabcYcaSiabdMgaPjabg2da9iabdYeamjabcQda6iabdkgaInaaBaaaleaacqWGRbWAaeqaaOWaaeWaaeaacqWGmbataiaawIcacaGLPaaacqGH9aqpcqWGHbqydaWgaaWcbaGaem4AaSMaemyraueabeaaaOqaaiabgcGiIiabigdaXiabgsMiJkabdMgaPjabgYda8iabdYeamjabcQda6iabdkgaInaaBaaaleaacqWGRbWAaeqaaOWaaeWaaeaacqWGPbqAaiaawIcacaGLPaaacqGH9aqpdaaeqbqaaiabdggaHnaaBaaaleaacqWGRbWAcqWGSbaBaeqaaOGaemyzau2aaSbaaSqaaiabdYgaSbqabaGcdaqadaqaaiabdIha4naaBaaaleaacqWGPbqAcqGHRaWkcqaIXaqmaeqaaaGccaGLOaGaayzkaaGaemOyai2aaSbaaSqaaiabdYgaSbqabaGcdaqadaqaaiabdMgaPjabgUcaRiabigdaXaGaayjkaiaawMcaaiaaxMaacaWLjaWaaeWaaeaacqaIXaqmcqaIWaamaiaawIcacaGLPaaaaSqaaiabdYgaSbqab0GaeyyeIuoaaOqaaiabdcfaqnaabmaabaacbeGae8hEaGNaeiiFaWhcciGae4hUdehacaGLOaGaayzkaaGaeyypa0ZaaabuaeaacqWGHbqydaWgaaWcbaGaemOqaiKaemiBaWgabeaakiabdwgaLnaaBaaaleaacqWGSbaBaeqaaOWaaeWaaeaacqWG4baEdaWgaaWcbaGaeGymaedabeaaaOGaayjkaiaawMcaaiabdkgaInaaBaaaleaacqWGSbaBaeqaaOWaaeWaaeaacqaIXaqmaiaawIcacaGLPaaaaSqaaiabdYgaSbqab0GaeyyeIuoaaaaa@873B@

The total likelihood computed by the Backward algorithm is exactly the same with the one computed by the Forward algorithm so it will rarely be used, however, the algorithm is essential for the posterior decoding, which we will consider later. Using exactly the same notation, we can write down the modified Backward algorithm:

#### Modified Backward algorithm

∀k,i=L:bkω(L)=akE∀1≤i<L:bkω(i)=∑laklel(xi+1)blω(i+1)dl(i+1)     (11)P(x,ω|θ)=∑laBlel(x1)blω(1)dl(1)
 MathType@MTEF@5@5@+=feaafiart1ev1aaatCvAUfKttLearuWrP9MDH5MBPbIqV92AaeXatLxBI9gBaebbnrfifHhDYfgasaacH8akY=wiFfYdH8Gipec8Eeeu0xXdbba9frFj0=OqFfea0dXdd9vqai=hGuQ8kuc9pgc9s8qqaq=dirpe0xb9q8qiLsFr0=vr0=vr0dc8meaabaqaciaacaGaaeqabaqabeGadaaakqaabeqaaiabgcGiIiabdUgaRjabcYcaSiabdMgaPjabg2da9iabdYeamjabcQda6iabdkgaInaaDaaaleaacqWGRbWAaeaaiiGacqWFjpWDaaGcdaqadaqaaiabdYeambGaayjkaiaawMcaaiabg2da9iabdggaHnaaBaaaleaacqWGRbWAcqWGfbqraeqaaaGcbaGaeyiaIiIaeGymaeJaeyizImQaemyAaKMaeyipaWJaemitaWKaeiOoaOJaemOyai2aa0baaSqaaiabdUgaRbqaaiab=L8a3baakmaabmaabaGaemyAaKgacaGLOaGaayzkaaGaeyypa0ZaaabuaeaacqWGHbqydaWgaaWcbaGaem4AaSMaemiBaWgabeaakiabdwgaLnaaBaaaleaacqWGSbaBaeqaaOWaaeWaaeaacqWG4baEdaWgaaWcbaGaemyAaKMaey4kaSIaeGymaedabeaaaOGaayjkaiaawMcaaiabdkgaInaaDaaaleaacqWGSbaBaeaacqWFjpWDaaGcdaqadaqaaiabdMgaPjabgUcaRiabigdaXaGaayjkaiaawMcaaiabdsgaKnaaBaaaleaacqWGSbaBaeqaaOWaaeWaaeaacqWGPbqAcqGHRaWkcqaIXaqmaiaawIcacaGLPaaacaWLjaGaaCzcamaabmaabaGaeGymaeJaeGymaedacaGLOaGaayzkaaaaleaacqWGSbaBaeqaniabggHiLdaakeaacqWGqbaudaqadaqaaGqabiab+Hha4jab+XcaSGGabiab9L8a3jabcYha8jab=H7aXbGaayjkaiaawMcaaiabg2da9maaqafabaGaemyyae2aaSbaaSqaaiabdkeacjabdYgaSbqabaGccqWGLbqzdaWgaaWcbaGaemiBaWgabeaakmaabmaabaGaemiEaG3aaSbaaSqaaiabigdaXaqabaaakiaawIcacaGLPaaacqWGIbGydaqhaaWcbaGaemiBaWgabaGae8xYdChaaOWaaeWaaeaacqaIXaqmaiaawIcacaGLPaaaaSqaaiabdYgaSbqab0GaeyyeIuoakiabdsgaKnaaBaaaleaacqWGSbaBaeqaaOWaaeWaaeaacqaIXaqmaiaawIcacaGLPaaaaaaa@9E17@

The likelihood computed by each one of the previous algorithms, is the joint likelihood of the sequences and the information given the model, which is always smaller than or equal to the likelihood computed by the standard algorithms:

P(x,ω|θ)=∑πP(x,ω,π|θ)=∑π∈ΠωP(x,π|θ)≤P(x|θ)     (12)
 MathType@MTEF@5@5@+=feaafiart1ev1aaatCvAUfKttLearuWrP9MDH5MBPbIqV92AaeXatLxBI9gBaebbnrfifHhDYfgasaacH8akY=wiFfYdH8Gipec8Eeeu0xXdbba9frFj0=OqFfea0dXdd9vqai=hGuQ8kuc9pgc9s8qqaq=dirpe0xb9q8qiLsFr0=vr0=vr0dc8meaabaqaciaacaGaaeqabaqabeGadaaakeaacqWGqbaudaqadaqaaGqabiab=Hha4jab=XcaSGGabiab+L8a3jabcYha8HGaciab9H7aXbGaayjkaiaawMcaaiabg2da9maaqafabaGaemiuaa1aaeWaaeaacqWF4baEcqWFSaalcqGFjpWDcqGFSaalcqqFapaCcqGG8baFcqqF4oqCaiaawIcacaGLPaaaaSqaaiab9b8aWbqab0GaeyyeIuoakiabg2da9maaqafabaGaemiuaa1aaeWaaeaacqWF4baEcqWFSaalcqqFapaCcqGG8baFcqqF4oqCaiaawIcacaGLPaaaaSqaaiab9b8aWjabgIGiolabfc6aqnaaBaaameaacqqFjpWDaeqaaaWcbeqdcqGHris5aOGaeyizImQaemiuaa1aaeWaaeaacqWF4baEcqGG8baFcqqF4oqCaiaawIcacaGLPaaacaWLjaGaaCzcamaabmaabaGaeGymaeJaeGOmaidacaGLOaGaayzkaaaaaa@69C8@

By using the Bayes theorem we can calculate the posterior probability of the information, given the sequences and the model, as follows:

P(ω|x,θ)=P(x,ω|θ)P(x|θ)     (13)
 MathType@MTEF@5@5@+=feaafiart1ev1aaatCvAUfKttLearuWrP9MDH5MBPbIqV92AaeXatLxBI9gBaebbnrfifHhDYfgasaacH8akY=wiFfYdH8Gipec8Eeeu0xXdbba9frFj0=OqFfea0dXdd9vqai=hGuQ8kuc9pgc9s8qqaq=dirpe0xb9q8qiLsFr0=vr0=vr0dc8meaabaqaciaacaGaaeqabaqabeGadaaakeaacqWGqbaudaqadaqaaGGabiab=L8a3jabcYha8Hqabiab+Hha4jabcYcaSGGaciab9H7aXbGaayjkaiaawMcaaiabg2da9maalaaabaGaemiuaa1aaeWaaeaacqGF4baEcqGFSaalcqWFjpWDcqGG8baFcqqF4oqCaiaawIcacaGLPaaaaeaacqWGqbaudaqadaqaaiab+Hha4jabcYha8jab9H7aXbGaayjkaiaawMcaaaaacaWLjaGaaCzcamaabmaabaGaeGymaeJaeG4mamdacaGLOaGaayzkaaaaaa@4DDF@

The last quantity informs us in to what extend our prior beliefs about the protein's topology have been changed when experimentally derived information emerges. If this probability is large, the information does not change our prior beliefs. However, when this probability is small enough, the experimentally derived information has changed a lot our prior beliefs (those based solely on the model), and thus we expect the prediction accuracy to be better.

### Decoding algorithms

Here we consider similar modifications to the algorithms used for decoding an HMM. The Viterbi algorithm is a dynamic programming algorithm that, on contrary to the Forward and Backward algorithms, finds the probability of the most probable path of states and not the total probability of all paths. With a backtracking step, the algorithm finally recovers the most probable path. The algorithm is conceptually similar to the Forward algorithm, where the consecutive summations are replaced by maximisations:

#### Viterbi algorithm

∀k≠B,i=0:uB(0)=1,uk(0)=0∀1≤i≤L:ul(i)=el(xi)max⁡k{uk(i−1)akl}     (14)P(x,πmax⁡|θ)=max⁡k{uk(L)akE}
 MathType@MTEF@5@5@+=feaafiart1ev1aaatCvAUfKttLearuWrP9MDH5MBPbIqV92AaeXatLxBI9gBaebbnrfifHhDYfgasaacH8akY=wiFfYdH8Gipec8Eeeu0xXdbba9frFj0=OqFfea0dXdd9vqai=hGuQ8kuc9pgc9s8qqaq=dirpe0xb9q8qiLsFr0=vr0=vr0dc8meaabaqaciaacaGaaeqabaqabeGadaaakqaabeqaaiabgcGiIiabdUgaRjabgcMi5kabdkeacjabcYcaSiabdMgaPjabg2da9iabicdaWiabcQda6iabdwha1naaBaaaleaacqWGcbGqaeqaaOWaaeWaaeaacqaIWaamaiaawIcacaGLPaaacqGH9aqpcqaIXaqmcqGGSaalcqWG1bqDdaWgaaWcbaGaem4AaSgabeaakmaabmaabaGaeGimaadacaGLOaGaayzkaaGaeyypa0JaeGimaadabaGaeyiaIiIaeGymaeJaeyizImQaemyAaKMaeyizImQaemitaWKaeiOoaOJaemyDau3aaSbaaSqaaiabdYgaSbqabaGcdaqadaqaaiabdMgaPbGaayjkaiaawMcaaiabg2da9iabdwgaLnaaBaaaleaacqWGSbaBaeqaaOWaaeWaaeaacqWG4baEdaWgaaWcbaGaemyAaKgabeaaaOGaayjkaiaawMcaamaaxababaGagiyBa0MaeiyyaeMaeiiEaGhaleaacqWGRbWAaeqaaOWaaiWaaeaacqWG1bqDdaWgaaWcbaGaem4AaSgabeaakmaabmaabaGaemyAaKMaeyOeI0IaeGymaedacaGLOaGaayzkaaGaemyyae2aaSbaaSqaaiabdUgaRjabdYgaSbqabaaakiaawUhacaGL9baacaWLjaGaaCzcamaabmaabaGaeGymaeJaeGinaqdacaGLOaGaayzkaaaabaGaemiuaa1aaeWaaeaaieqacqWF4baEcqGGSaaliiGacqGFapaCdaahaaWcbeqaaiGbc2gaTjabcggaHjabcIha4baakiabcYha8jab+H7aXbGaayjkaiaawMcaaiabg2da9maaxababaGagiyBa0MaeiyyaeMaeiiEaGhaleaacqWGRbWAaeqaaOWaaiWaaeaacqWG1bqDdaWgaaWcbaGaem4AaSgabeaakmaabmaabaGaemitaWeacaGLOaGaayzkaaGaemyyae2aaSbaaSqaaiabdUgaRjabdweafbqabaaakiaawUhacaGL9baaaaaa@9726@

Here, *π*^*max *^is the most probable path of states and *P*(**x**, *π*^*max*^|*θ*) its probability, with *P*(**x**,*π*^max^|*θ*)≤*P*(**x**|*θ*). In the modification to the Viterbi algorithm, sketched below, we use exactly the same constrains in order to force the algorithm to consider only the paths compatible with the information **ω**. In this case, the joint probability of the best path and the information, is denoted *P*(**x**, *π*^ω^|*θ*).

#### Modified Viterbi algorithm

∀k≠B,i=0:uBω(0)=1,ukω(0)=0∀1≤i≤L:ulω(i)=dl(i)el(xi)max⁡k{ukω(i−1)akl}     (15)P(x,ω,πω|θ)=max⁡k{ukω(L)akE}
 MathType@MTEF@5@5@+=feaafiart1ev1aaatCvAUfKttLearuWrP9MDH5MBPbIqV92AaeXatLxBI9gBaebbnrfifHhDYfgasaacH8akY=wiFfYdH8Gipec8Eeeu0xXdbba9frFj0=OqFfea0dXdd9vqai=hGuQ8kuc9pgc9s8qqaq=dirpe0xb9q8qiLsFr0=vr0=vr0dc8meaabaqaciaacaGaaeqabaqabeGadaaakqaabeqaaiabgcGiIiabdUgaRjabgcMi5kabdkeacjabcYcaSiabdMgaPjabg2da9iabicdaWiabcQda6iabdwha1naaDaaaleaacqWGcbGqaeaaiiGacqWFjpWDaaGcdaqadaqaaiabicdaWaGaayjkaiaawMcaaiabg2da9iabigdaXiabcYcaSiabdwha1naaDaaaleaacqWGRbWAaeaacqWFjpWDaaGcdaqadaqaaiabicdaWaGaayjkaiaawMcaaiabg2da9iabicdaWaqaaiabgcGiIiabigdaXiabgsMiJkabdMgaPjabgsMiJkabdYeamjabcQda6iabdwha1naaDaaaleaacqWGSbaBaeaacqWFjpWDaaGcdaqadaqaaiabdMgaPbGaayjkaiaawMcaaiabg2da9iabdsgaKnaaBaaaleaacqWGSbaBaeqaaOWaaeWaaeaacqWGPbqAaiaawIcacaGLPaaacqWGLbqzdaWgaaWcbaGaemiBaWgabeaakmaabmaabaGaemiEaG3aaSbaaSqaaiabdMgaPbqabaaakiaawIcacaGLPaaadaWfqaqaaiGbc2gaTjabcggaHjabcIha4bWcbaGaem4AaSgabeaakmaacmaabaGaemyDau3aa0baaSqaaiabdUgaRbqaaiab=L8a3baakmaabmaabaGaemyAaKMaeyOeI0IaeGymaedacaGLOaGaayzkaaGaemyyae2aaSbaaSqaaiabdUgaRjabdYgaSbqabaaakiaawUhacaGL9baacaWLjaGaaCzcamaabmaabaGaeGymaeJaeGynaudacaGLOaGaayzkaaaabaGaemiuaa1aaeWaaeaaieqacqGF4baEcqGGSaaliiqacqqFjpWDcqGGSaalcqWFapaCdaahaaWcbeqaaiab=L8a3baakiabcYha8jab=H7aXbGaayjkaiaawMcaaiabg2da9maaxababaGagiyBa0MaeiyyaeMaeiiEaGhaleaacqWGRbWAaeqaaOWaaiWaaeaacqWG1bqDdaqhaaWcbaGaem4AaSgabaGae8xYdChaaOWaaeWaaeaacqWGmbataiaawIcacaGLPaaacqWGHbqydaWgaaWcbaGaem4AaSMaemyraueabeaaaOGaay5Eaiaaw2haaaaaaa@A635@

The 1-best decoding [33], is a modification of the N-best decoding method, proposed earlier for speech recognition [52]. The algorithm is a heuristic that tries to find the most probable path of labels **y**^max ^of a sequence instead of the most probable path of states. For each position *i *in the sequence, keeps track of all the possible active hypotheses *h*_*i-1 *_that consist of all the possible sequence of labels up to that point. Afterwards, for each state *l*, propagates these hypotheses, appending each one of the possible labels *y*_*i*_, and picks up the best, until the end of the sequence. In contrast to the Viterbi algorithm, 1-best does not need a Traceback procedure:

#### 1-best algorithm

i=1:γl(h1)=aBlel(x1)∀1<i≤L:γl(hiyi)=el(xi)∑kγk(hi−1)akl     (16)P(x,ymax⁡|θ)=∑kγk(hL)akE
 MathType@MTEF@5@5@+=feaafiart1ev1aaatCvAUfKttLearuWrP9MDH5MBPbIqV92AaeXatLxBI9gBaebbnrfifHhDYfgasaacH8akY=wiFfYdH8Gipec8Eeeu0xXdbba9frFj0=OqFfea0dXdd9vqai=hGuQ8kuc9pgc9s8qqaq=dirpe0xb9q8qiLsFr0=vr0=vr0dc8meaabaqaciaacaGaaeqabaqabeGadaaakqaabeqaaiabdMgaPjabg2da9iabigdaXiabcQda6GGaciab=n7aNnaaBaaaleaacqWGSbaBaeqaaOWaaeWaaeaacqWGObaAdaWgaaWcbaGaeGymaedabeaaaOGaayjkaiaawMcaaiabg2da9iabdggaHnaaBaaaleaacqWGcbGqcqWGSbaBaeqaaOGaemyzau2aaSbaaSqaaiabdYgaSbqabaGcdaqadaqaaiabdIha4naaBaaaleaacqaIXaqmaeqaaaGccaGLOaGaayzkaaaabaGaeyiaIiIaeGymaeJaeyipaWJaemyAaKMaeyizImQaemitaWKaeiOoaOJae83SdC2aaSbaaSqaaiabdYgaSbqabaGcdaqadaqaaiabdIgaOnaaBaaaleaacqWGPbqAaeqaaOGaemyEaK3aaSbaaSqaaiabdMgaPbqabaaakiaawIcacaGLPaaacqGH9aqpcqWGLbqzdaWgaaWcbaGaemiBaWgabeaakmaabmaabaGaemiEaG3aaSbaaSqaaiabdMgaPbqabaaakiaawIcacaGLPaaadaaeqbqaaiab=n7aNnaaBaaaleaacqWGRbWAaeqaaOWaaeWaaeaacqWGObaAdaWgaaWcbaGaemyAaKMaeyOeI0IaeGymaedabeaaaOGaayjkaiaawMcaaiabdggaHnaaBaaaleaacqWGRbWAcqWGSbaBaeqaaaqaaiabdUgaRbqab0GaeyyeIuoakiaaxMaacaWLjaWaaeWaaeaacqaIXaqmcqaI2aGnaiaawIcacaGLPaaaaeaacqWGqbaudaqadaqaaGqabiab+Hha4jabcYcaSiab+Lha5naaCaaaleqabaGagiyBa0MaeiyyaeMaeiiEaGhaaOGaeiiFaWNae8hUdehacaGLOaGaayzkaaGaeyypa0ZaaabuaeaacqWFZoWzdaWgaaWcbaGaem4AaSgabeaakmaabmaabaGaemiAaG2aaSbaaSqaaiabdYeambqabaaakiaawIcacaGLPaaacqWGHbqydaWgaaWcbaGaem4AaSMaemyraueabeaaaeaacqWGRbWAaeqaniabggHiLdaaaaa@9361@

Although this algorithm is a heuristic and no guarantee exists for finding the most probable labelling, the probability of the best reported labelling is always larger than, or equal to, the probability of the best path, and always smaller than, or equal to, the total probability of the sequence, since a lot of allowed paths are contributing to this, thus: *P*(**x**,*π*^max ^| *θ*)≤*P*(**x**,**y**^max ^| *θ*)≤*P*(**x **| *θ*). Obviously, we just have to set once again the intermediate variable *γ *equal to zero for the states *k *and residues *i *that are not in agreement with the prior information **ω. **In Viterbi and 1-best decoding, similar measures with that of Equation (13) can be obtained.

#### Modified 1-best algorithm

i=1:γlω(h1)=aBlel(x1)∀1≤i≤L:γlω(hiyi)=dl(i)el(xi)∑kγkω(hi−1)akl     (17)P(x,yω|θ)=∑kγkω(hL)akE
 MathType@MTEF@5@5@+=feaafiart1ev1aaatCvAUfKttLearuWrP9MDH5MBPbIqV92AaeXatLxBI9gBaebbnrfifHhDYfgasaacH8akY=wiFfYdH8Gipec8Eeeu0xXdbba9frFj0=OqFfea0dXdd9vqai=hGuQ8kuc9pgc9s8qqaq=dirpe0xb9q8qiLsFr0=vr0=vr0dc8meaabaqaciaacaGaaeqabaqabeGadaaakqaabeqaaiabdMgaPjabg2da9iabigdaXiabcQda6GGaciab=n7aNnaaDaaaleaacqWGSbaBaeaacqWFjpWDaaGcdaqadaqaaiabdIgaOnaaBaaaleaacqaIXaqmaeqaaaGccaGLOaGaayzkaaGaeyypa0Jaemyyae2aaSbaaSqaaiabdkeacjabdYgaSbqabaGccqWGLbqzdaWgaaWcbaGaemiBaWgabeaakmaabmaabaGaemiEaG3aaSbaaSqaaiabigdaXaqabaaakiaawIcacaGLPaaaaeaacqGHaiIicqaIXaqmcqGHKjYOcqWGPbqAcqGHKjYOcqWGmbatcqGG6aGocqWFZoWzdaqhaaWcbaGaemiBaWgabaGae8xYdChaaOWaaeWaaeaacqWGObaAdaWgaaWcbaGaemyAaKgabeaakiabdMha5naaBaaaleaacqWGPbqAaeqaaaGccaGLOaGaayzkaaGaeyypa0Jaemizaq2aaSbaaSqaaiabdYgaSbqabaGcdaqadaqaaiabdMgaPbGaayjkaiaawMcaaiabdwgaLnaaBaaaleaacqWGSbaBaeqaaOWaaeWaaeaacqWG4baEdaWgaaWcbaGaemyAaKgabeaaaOGaayjkaiaawMcaamaaqafabaGae83SdC2aa0baaSqaaiabdUgaRbqaaiab=L8a3baakmaabmaabaGaemiAaG2aaSbaaSqaaiabdMgaPjabgkHiTiabigdaXaqabaaakiaawIcacaGLPaaacqWGHbqydaWgaaWcbaGaem4AaSMaemiBaWgabeaaaeaacqWGRbWAaeqaniabggHiLdGccaWLjaGaaCzcamaabmaabaGaeGymaeJaeG4naCdacaGLOaGaayzkaaaabaGaemiuaa1aaeWaaeaaieqacqGF4baEcqGGSaalcqGF5bqEdaahaaWcbeqaaiab=L8a3baakiabcYha8jab=H7aXbGaayjkaiaawMcaaiabg2da9maaqafabaGae83SdC2aa0baaSqaaiabdUgaRbqaaiab=L8a3baakmaabmaabaGaemiAaG2aaSbaaSqaaiabdYeambqabaaakiaawIcacaGLPaaacqWGHbqydaWgaaWcbaGaem4AaSMaemyraueabeaaaeaacqWGRbWAaeqaniabggHiLdaaaaa@9EA6@

#### Posterior decoding

The posterior decoding method is based on calculating the "*a-posteriori*" probability of a residue *x*_*i *_to be emitted by a state *k*, having observed the whole sequence **x**. This probability is calculated using the forward and backward variables *f*, *b*:

P(πi=k|x,θ)=fk(i)bk(i)P(x,θ)     (18)
 MathType@MTEF@5@5@+=feaafiart1ev1aaatCvAUfKttLearuWrP9MDH5MBPbIqV92AaeXatLxBI9gBaebbnrfifHhDYfgasaacH8akY=wiFfYdH8Gipec8Eeeu0xXdbba9frFj0=OqFfea0dXdd9vqai=hGuQ8kuc9pgc9s8qqaq=dirpe0xb9q8qiLsFr0=vr0=vr0dc8meaabaqaciaacaGaaeqabaqabeGadaaakeaacqWGqbaudaqadaqaaGGaciab=b8aWnaaBaaaleaacqWGPbqAaeqaaOGaeyypa0Jaem4AaSMaeiiFaWhcbeGae4hEaGNaeiilaWIae8hUdehacaGLOaGaayzkaaGaeyypa0ZaaSaaaeaacqWGMbGzdaWgaaWcbaGaem4AaSgabeaakmaabmaabaGaemyAaKgacaGLOaGaayzkaaGaemOyai2aaSbaaSqaaiabdUgaRbqabaGcdaqadaqaaiabdMgaPbGaayjkaiaawMcaaaqaaiabdcfaqnaabmaabaGae4hEaGNaeiilaWIae8hUdehacaGLOaGaayzkaaaaaiaaxMaacaWLjaWaaeWaaeaacqaIXaqmcqaI4aaoaiaawIcacaGLPaaaaaa@52C3@

In cases of states sharing the same labelling, we usually sum for each position *i *along the sequence, the posterior probabilities of the states with the same labelling *c *[2], calculating thus the quantity called Posterior Label Probability (PLP) in [36]:

gc(i)=P(yi=c|x,θ)=∑kP(πi=k|x,θ)λk(c)     (19)
 MathType@MTEF@5@5@+=feaafiart1ev1aaatCvAUfKttLearuWrP9MDH5MBPbIqV92AaeXatLxBI9gBaebbnrfifHhDYfgasaacH8akY=wiFfYdH8Gipec8Eeeu0xXdbba9frFj0=OqFfea0dXdd9vqai=hGuQ8kuc9pgc9s8qqaq=dirpe0xb9q8qiLsFr0=vr0=vr0dc8meaabaqaciaacaGaaeqabaqabeGadaaakeaacqWGNbWzdaWgaaWcbaGaem4yamgabeaakmaabmaabaGaemyAaKgacaGLOaGaayzkaaGaeyypa0Jaemiuaa1aaeWaaeaacqWG5bqEdaWgaaWcbaGaemyAaKgabeaakiabg2da9iabdogaJjabcYha8Hqabiab=Hha4jabcYcaSGGaciab+H7aXbGaayjkaiaawMcaaiabg2da9maaqafabaGaemiuaa1aaeWaaeaacqGFapaCdaWgaaWcbaGaemyAaKgabeaakiabg2da9iabdUgaRjabcYha8jab=Hha4jabcYcaSiab+H7aXbGaayjkaiaawMcaaiab+T7aSnaaBaaaleaacqWGRbWAaeqaaOWaaeWaaeaacqWGJbWyaiaawIcacaGLPaaaaSqaaiabdUgaRbqab0GaeyyeIuoakiaaxMaacaWLjaWaaeWaaeaacqaIXaqmcqaI5aqoaiaawIcacaGLPaaaaaa@5E6E@

A naïve approach would be to consider as a path, the sequence of labels that maximises this probability for each position along the sequence. Such an approach for the posterior decoding is capable of yielding paths inconsistent with the model, for instance, it could produce a path according to which an intracellular loop (I) is followed by a segment of residues predicted to be extracellular (O). However, when these posterior probabilities are filtered properly, in many situations have been proved to yield results with better prediction accuracy [34,53]. The Dynamic programming solution to the problem of detecting the optimal location and length of *n *transmembrane segments in a sequence of *m *residues, is to divide the problem in *n *smaller problems, thus dealing with each transmembrane segment separately [37]. If we denote by *s*^*il *^the sum of the posterior label probabilities of transmembrane segments, for a segment of length *l *at position *i *of a sequence, then the overall score Sji
 MathType@MTEF@5@5@+=feaafiart1ev1aaatCvAUfKttLearuWrP9MDH5MBPbIqV92AaeXatLxBI9gBaebbnrfifHhDYfgasaacH8akY=wiFfYdH8Gipec8Eeeu0xXdbba9frFj0=OqFfea0dXdd9vqai=hGuQ8kuc9pgc9s8qqaq=dirpe0xb9q8qiLsFr0=vr0=vr0dc8meaabaqaciaacaGaaeqabaqabeGadaaakeaacqWGtbWudaqhaaWcbaGaemOAaOgabaGaemyAaKgaaaaa@30C0@(*i*: 1, 2,...*n*; *j*:1, 2,...,*m*), will be calculated by a recursive relation:

Sji=max⁡l=lmin⁡→lmax⁡{sjil+max⁡k=1+l+A→n{Sj−1k}}     (20)
 MathType@MTEF@5@5@+=feaafiart1ev1aaatCvAUfKttLearuWrP9MDH5MBPbIqV92AaeXatLxBI9gBaebbnrfifHhDYfgasaacH8akY=wiFfYdH8Gipec8Eeeu0xXdbba9frFj0=OqFfea0dXdd9vqai=hGuQ8kuc9pgc9s8qqaq=dirpe0xb9q8qiLsFr0=vr0=vr0dc8meaabaqaciaacaGaaeqabaqabeGadaaakeaacqWGtbWudaqhaaWcbaGaemOAaOgabaGaemyAaKgaaOGaeyypa0ZaaCbeaeaacyGGTbqBcqGGHbqycqGG4baEaSqaaiabdYgaSjabg2da9iabdYgaSnaaBaaameaacyGGTbqBcqGGPbqAcqGGUbGBaeqaaSGaeyOKH4QaemiBaW2aaSbaaWqaaiGbc2gaTjabcggaHjabcIha4bqabaaaleqaaOWaaiWaaeaacqWGZbWCdaqhaaWcbaGaemOAaOgabaGaemyAaKMaemiBaWgaaOGaey4kaSYaaCbeaeaacyGGTbqBcqGGHbqycqGG4baEaSqaaiabdUgaRjabg2da9iabigdaXiabgUcaRiabdYgaSjabgUcaRiabdgeabjabgkziUkabd6gaUbqabaGcdaGadaqaaiabdofatnaaDaaaleaacqWGQbGAcqGHsislcqaIXaqmaeaacqWGRbWAaaaakiaawUhacaGL9baaaiaawUhacaGL9baacaWLjaGaaCzcamaabmaabaGaeGOmaiJaeGimaadacaGLOaGaayzkaaaaaa@6AF6@

where *j *is the total number of transmembrane segments, *l*_*min *_and *l*_*max *_the minimum and maximum allowed lengths for the transmembrane segments respectively, and *A *the minimum allowed length of a turn.

With the use of the modified Forward and Backward algorithms described above, we can similarly calculate the posterior probability of a residue *i *to be emitted by a state *k*, given the sequence **x **and the information **ω**, as follows:

P(πi=k|x,ω,θ)=fkω(i)bkω(i)P(x,ω,θ),     (21)
 MathType@MTEF@5@5@+=feaafiart1ev1aaatCvAUfKttLearuWrP9MDH5MBPbIqV92AaeXatLxBI9gBaebbnrfifHhDYfgasaacH8akY=wiFfYdH8Gipec8Eeeu0xXdbba9frFj0=OqFfea0dXdd9vqai=hGuQ8kuc9pgc9s8qqaq=dirpe0xb9q8qiLsFr0=vr0=vr0dc8meaabaqaciaacaGaaeqabaqabeGadaaakeaacqWGqbaudaqadaqaaGGaciab=b8aWnaaBaaaleaacqWGPbqAaeqaaOGaeyypa0Jaem4AaSMaeiiFaWhcbeGae4hEaGNaeiilaWccceGae0xYdCNaeiilaWIae8hUdehacaGLOaGaayzkaaGaeyypa0ZaaSaaaeaacqWGMbGzdaqhaaWcbaGaem4AaSgabaGae8xYdChaaOWaaeWaaeaacqWGPbqAaiaawIcacaGLPaaacqWGIbGydaqhaaWcbaGaem4AaSgabaGae8xYdChaaOWaaeWaaeaacqWGPbqAaiaawIcacaGLPaaaaeaacqWGqbaudaqadaqaaiab+Hha4jabcYcaSiab9L8a3jabcYcaSiab=H7aXbGaayjkaiaawMcaaaaacqGGSaalcaWLjaGaaCzcamaabmaabaGaeGOmaiJaeGymaedacaGLOaGaayzkaaaaaa@5C80@

And finally, we can calculate the PLP for the label *c *at position *i *given the information:

gclω(i)=P(yi=cl|x,ω,θ)=∑kP(πi=k|x,ω,θ)λk(cl)     (22)
 MathType@MTEF@5@5@+=feaafiart1ev1aaatCvAUfKttLearuWrP9MDH5MBPbIqV92AaeXatLxBI9gBaebbnrfifHhDYfgasaacH8akY=wiFfYdH8Gipec8Eeeu0xXdbba9frFj0=OqFfea0dXdd9vqai=hGuQ8kuc9pgc9s8qqaq=dirpe0xb9q8qiLsFr0=vr0=vr0dc8meaabaqaciaacaGaaeqabaqabeGadaaakeaacqWGNbWzdaqhaaWcbaGaem4yam2aaWbaaWqabeaacqWGSbaBaaaaleaaiiGacqWFjpWDaaGcdaqadaqaaiabdMgaPbGaayjkaiaawMcaaiabg2da9iabdcfaqnaabmaabaGaemyEaK3aaSbaaSqaaiabdMgaPbqabaGccqGH9aqpcqWGJbWydaahaaWcbeqaaiabdYgaSbaakiabcYha8Hqabiab+Hha4jabcYcaSGGabiab9L8a3jabcYcaSiab=H7aXbGaayjkaiaawMcaaiabg2da9maaqafabaGaemiuaa1aaeWaaeaacqWFapaCdaWgaaWcbaGaemyAaKgabeaakiabg2da9iabdUgaRjabcYha8jab+Hha4jabcYcaSiab9L8a3jabcYcaSiab=H7aXbGaayjkaiaawMcaaiab=T7aSnaaBaaaleaacqWGRbWAaeqaaOWaaeWaaeaacqWGJbWydaahaaWcbeqaaiabdYgaSbaaaOGaayjkaiaawMcaaaWcbaGaem4AaSgabeqdcqGHris5aOGaaCzcaiaaxMaadaqadaqaaiabikdaYiabikdaYaGaayjkaiaawMcaaaaa@6A4E@

Even though the PLPs obtained this way are consistent with the prior knowledge, the dynamic programming algorithm described in Equation (20), *is not guaranteed *to yield a consistent result, and thus more refined solutions should be pursued.

#### Posterior-Viterbi decoding

Recently, Fariselli and co-workers have provided a decoding algorithm that combines elements of the Viterbi and the posterior decoding [35]. The algorithm finds the path *π*^*PV*^, such as

πPV=arg⁡max⁡π∈Πp∏i=1LP(πi|x)     (23)
 MathType@MTEF@5@5@+=feaafiart1ev1aaatCvAUfKttLearuWrP9MDH5MBPbIqV92AaeXatLxBI9gBaebbnrfifHhDYfgasaacH8akY=wiFfYdH8Gipec8Eeeu0xXdbba9frFj0=OqFfea0dXdd9vqai=hGuQ8kuc9pgc9s8qqaq=dirpe0xb9q8qiLsFr0=vr0=vr0dc8meaabaqaciaacaGaaeqabaqabeGadaaakeaaiiGacqWFapaCdaahaaWcbeqaaiabdcfaqjabdAfawbaakiabg2da9maaxababaGagiyyaeMaeiOCaiNaei4zaCMagiyBa0MaeiyyaeMaeiiEaGhaleaacqWFapaCcqGHiiIZcqqHGoaudaWgaaadbaGaemiCaahabeaaaSqabaGcdaqeWbqaaiabdcfaqnaabmaabaGae8hWda3aaSbaaSqaaiabdMgaPbqabaGccqGG8baFieqacqGF4baEaiaawIcacaGLPaaaaSqaaiabdMgaPjabg2da9iabigdaXaqaaiabdYeambqdcqGHpis1aOGaaCzcaiaaxMaadaqadaqaaiabikdaYiabiodaZaGaayjkaiaawMcaaaaa@5536@

where Π_*p *_is the allowed paths through the model and *P*(*π*_*i *_= *k*|**x**) is the posterior probability for the state *k *at position *i*. To define the allowed paths, we need a delta function, which takes value 1 if the transition *α*_*kl *_is an allowed one and 0 otherwise. Thus:

δ(k,l)={1,if akl>00,otherwise     (24)
 MathType@MTEF@5@5@+=feaafiart1ev1aaatCvAUfKttLearuWrP9MDH5MBPbIqV92AaeXatLxBI9gBaebbnrfifHhDYfgasaacH8akY=wiFfYdH8Gipec8Eeeu0xXdbba9frFj0=OqFfea0dXdd9vqai=hGuQ8kuc9pgc9s8qqaq=dirpe0xb9q8qiLsFr0=vr0=vr0dc8meaabaqaciaacaGaaeqabaqabeGadaaakeaaiiGacqWF0oazdaqadaqaaiabdUgaRjabcYcaSiabdYgaSbGaayjkaiaawMcaaiabg2da9maaceaabaqbaeqabiqaaaqaaiabigdaXiabcYcaSiabbMgaPjabbAgaMjabbccaGiabdggaHnaaBaaaleaacqWGRbWAcqWGSbaBaeqaaOGaeyOpa4JaeGimaadabaGaeGimaaJaeiilaWIaee4Ba8MaeeiDaqNaeeiAaGMaeeyzauMaeeOCaiNaee4DaCNaeeyAaKMaee4CamNaeeyzaugaaiaaxMaacaWLjaWaaeWaaeaacqaIYaGmcqaI0aanaiaawIcacaGLPaaaaiaawUhaaaaa@541E@

Finally, the optimal allowed-posterior path *π*^*PV*^, is denoted by:

πPV=arg⁡max⁡π∏i=1Lδ(πi,πi+1)P(πi|x)     (25)
 MathType@MTEF@5@5@+=feaafiart1ev1aaatCvAUfKttLearuWrP9MDH5MBPbIqV92AaeXatLxBI9gBaebbnrfifHhDYfgasaacH8akY=wiFfYdH8Gipec8Eeeu0xXdbba9frFj0=OqFfea0dXdd9vqai=hGuQ8kuc9pgc9s8qqaq=dirpe0xb9q8qiLsFr0=vr0=vr0dc8meaabaqaciaacaGaaeqabaqabeGadaaakeaaiiGacqWFapaCdaahaaWcbeqaaiabdcfaqjabdAfawbaakiabg2da9maaxababaGagiyyaeMaeiOCaiNaei4zaCMagiyBa0MaeiyyaeMaeiiEaGhaleaacqWFapaCaeqaaOWaaebCaeaacqWF0oazdaqadaqaaiab=b8aWnaaBaaaleaacqWGPbqAaeqaaOGaeiilaWIae8hWda3aaSbaaSqaaiabdMgaPjabgUcaRiabigdaXaqabaaakiaawIcacaGLPaaacqWGqbaudaqadaqaaiab=b8aWnaaBaaaleaacqWGPbqAaeqaaOGaeiiFaWhcbeGae4hEaGhacaGLOaGaayzkaaaaleaacqWGPbqAcqGH9aqpcqaIXaqmaeaacqWGmbata0Gaey4dIunakiaaxMaacaWLjaWaaeWaaeaacqaIYaGmcqaI1aqnaiaawIcacaGLPaaaaaa@5D04@

The Posterior-Viterbi decoding algorithm performs essentially a Viterbi-like decoding, using the Posterior probabilities instead of the emissions, and the allowed paths given by the above mentioned delta function instead of the transitions.

#### Posterior-Viterbi algorithm

∀k≠B,i=0:uB(0)=1,uk(0)=0∀1≤i≤L:ul(i)=P(πi=l|x,θ)max⁡k{uk(i−1)δ(k,l)}     (26)P(x,πPV|θ)=max⁡k{uk(L)δ(k,E)}
 MathType@MTEF@5@5@+=feaafiart1ev1aaatCvAUfKttLearuWrP9MDH5MBPbIqV92AaeXatLxBI9gBaebbnrfifHhDYfgasaacH8akY=wiFfYdH8Gipec8Eeeu0xXdbba9frFj0=OqFfea0dXdd9vqai=hGuQ8kuc9pgc9s8qqaq=dirpe0xb9q8qiLsFr0=vr0=vr0dc8meaabaqaciaacaGaaeqabaqabeGadaaakqaabeqaaiabgcGiIiabdUgaRjabgcMi5kabdkeacjabcYcaSiabdMgaPjabg2da9iabicdaWiabcQda6iabdwha1naaBaaaleaacqWGcbGqaeqaaOWaaeWaaeaacqaIWaamaiaawIcacaGLPaaacqGH9aqpcqaIXaqmcqGGSaalcqWG1bqDdaWgaaWcbaGaem4AaSgabeaakmaabmaabaGaeGimaadacaGLOaGaayzkaaGaeyypa0JaeGimaadabaGaeyiaIiIaeGymaeJaeyizImQaemyAaKMaeyizImQaemitaWKaeiOoaOJaemyDau3aaSbaaSqaaiabdYgaSbqabaGcdaqadaqaaiabdMgaPbGaayjkaiaawMcaaiabg2da9iabdcfaqnaabmaabaacciGae8hWda3aaSbaaSqaaiabdMgaPbqabaGccqGH9aqpcqWGSbaBcqGG8baFieqacqGF4baEcqGGSaalcqWF4oqCaiaawIcacaGLPaaadaWfqaqaaiGbc2gaTjabcggaHjabcIha4bWcbaGaem4AaSgabeaakmaacmaabaGaemyDau3aaSbaaSqaaiabdUgaRbqabaGcdaqadaqaaiabdMgaPjabgkHiTiabigdaXaGaayjkaiaawMcaaiab=r7aKnaabmaabaGaem4AaSMaeiilaWIaemiBaWgacaGLOaGaayzkaaaacaGL7bGaayzFaaGaaCzcaiaaxMaadaqadaqaaiabikdaYiabiAda2aGaayjkaiaawMcaaaqaaiabdcfaqnaabmaabaGae4hEaGNaeiilaWIae8hWda3aaWbaaSqabeaacqWGqbaucqWGwbGvaaGccqGG8baFcqWF4oqCaiaawIcacaGLPaaacqGH9aqpdaWfqaqaaiGbc2gaTjabcggaHjabcIha4bWcbaGaem4AaSgabeaakmaacmaabaGaemyDau3aaSbaaSqaaiabdUgaRbqabaGcdaqadaqaaiabdYeambGaayjkaiaawMcaaiab=r7aKnaabmaabaGaem4AaSMaeiilaWIaemyraueacaGLOaGaayzkaaaacaGL7bGaayzFaaaaaaa@A0DF@

Similar to the Viterbi algorithm, the modified Posterior-Viterbi is as follows:

#### Modified Posterior-Viterbi algorithm

∀k≠B,i=0:uBω(0)=1,ukω(0)=0∀1≤i≤L:ulω(i)=dl(i)P(πi=l|x,ω,θ)max⁡k{ukω(i−1)δ(k,l)}     (27)P(x,ω,πPV,ω|θ)=max⁡k{ukω(L)δ(k,E)}
 MathType@MTEF@5@5@+=feaafiart1ev1aaatCvAUfKttLearuWrP9MDH5MBPbIqV92AaeXatLxBI9gBaebbnrfifHhDYfgasaacH8akY=wiFfYdH8Gipec8Eeeu0xXdbba9frFj0=OqFfea0dXdd9vqai=hGuQ8kuc9pgc9s8qqaq=dirpe0xb9q8qiLsFr0=vr0=vr0dc8meaabaqaciaacaGaaeqabaqabeGadaaakqaabeqaaiabgcGiIiabdUgaRjabgcMi5kabdkeacjabcYcaSiabdMgaPjabg2da9iabicdaWiabcQda6iabdwha1naaDaaaleaacqWGcbGqaeaaiiGacqWFjpWDaaGcdaqadaqaaiabicdaWaGaayjkaiaawMcaaiabg2da9iabigdaXiabcYcaSiabdwha1naaDaaaleaacqWGRbWAaeaacqWFjpWDaaGcdaqadaqaaiabicdaWaGaayjkaiaawMcaaiabg2da9iabicdaWaqaaiabgcGiIiabigdaXiabgsMiJkabdMgaPjabgsMiJkabdYeamjabcQda6iabdwha1naaDaaaleaacqWGSbaBaeaacqWFjpWDaaGcdaqadaqaaiabdMgaPbGaayjkaiaawMcaaiabg2da9iabdsgaKnaaBaaaleaacqWGSbaBaeqaaOWaaeWaaeaacqWGPbqAaiaawIcacaGLPaaacqWGqbaudaqadaqaaiab=b8aWnaaBaaaleaacqWGPbqAaeqaaOGaeyypa0JaemiBaWMaeiiFaWhcbeGae4hEaGNaeiilaWccceGae0xYdCNaeiilaWIae8hUdehacaGLOaGaayzkaaWaaCbeaeaacyGGTbqBcqGGHbqycqGG4baEaSqaaiabdUgaRbqabaGcdaGadaqaaiabdwha1naaDaaaleaacqWGRbWAaeaacqWFjpWDaaGcdaqadaqaaiabdMgaPjabgkHiTiabigdaXaGaayjkaiaawMcaaiab=r7aKnaabmaabaGaem4AaSMaeiilaWIaemiBaWgacaGLOaGaayzkaaaacaGL7bGaayzFaaGaaCzcaiaaxMaadaqadaqaaiabikdaYiabiEda3aGaayjkaiaawMcaaaqaaiabdcfaqnaabmaabaGae4hEaGNaeiilaWIae0xYdCNaeiilaWIae8hWda3aaWbaaSqabeaacqWGqbaucqWGwbGvcqGGSaalcqWFjpWDaaGccqGG8baFcqWF4oqCaiaawIcacaGLPaaacqGH9aqpdaWfqaqaaiGbc2gaTjabcggaHjabcIha4bWcbaGaem4AaSgabeaakmaacmaabaGaemyDau3aa0baaSqaaiabdUgaRbqaaiab=L8a3baakmaabmaabaGaemitaWeacaGLOaGaayzkaaGae8hTdq2aaeWaaeaacqWGRbWAcqGGSaalcqWGfbqraiaawIcacaGLPaaaaiaawUhacaGL9baaaaaa@B799@

where, instead of the standard posterior probabilities we used those computed by the modified Forward and Backward algorithms in Equation (10).

#### Optimal Accuracy Posterior Decoding

Finally, Kall and coworkers presented a very similar algorithm, the Optimal Accuracy Posterior Decoder [36]. The algorithm sums for each position in the sequence the posterior label probabilities (PLPs), using Equation (19), and by using the allowed transitions defined by Equation (24), calculates in a Viterbi-like manner the optimal sequence of labels that maximises the quantity:

πOAPD=arg⁡max⁡π∑i=1L{δ(πi,πi+1)(∑kP(πi|x)λk(c))}     (28)
 MathType@MTEF@5@5@+=feaafiart1ev1aaatCvAUfKttLearuWrP9MDH5MBPbIqV92AaeXatLxBI9gBaebbnrfifHhDYfgasaacH8akY=wiFfYdH8Gipec8Eeeu0xXdbba9frFj0=OqFfea0dXdd9vqai=hGuQ8kuc9pgc9s8qqaq=dirpe0xb9q8qiLsFr0=vr0=vr0dc8meaabaqaciaacaGaaeqabaqabeGadaaakeaaiiGacqWFapaCdaahaaWcbeqaaiabd+eapjabdgeabjabdcfaqjabdseaebaakiabg2da9maaxababaGagiyyaeMaeiOCaiNaei4zaCMagiyBa0MaeiyyaeMaeiiEaGhaleaacqWFapaCaeqaaOWaaabCaeaadaGadaqaaiab=r7aKnaabmaabaGae8hWda3aaSbaaSqaaiabdMgaPbqabaGccqGGSaalcqWFapaCdaWgaaWcbaGaemyAaKMaey4kaSIaeGymaedabeaaaOGaayjkaiaawMcaamaabmaabaWaaabuaeaacqWGqbaudaqadaqaaiab=b8aWnaaBaaaleaacqWGPbqAaeqaaOGaeiiFaWhcbeGae4hEaGhacaGLOaGaayzkaaGae83UdW2aaSbaaSqaaiabdUgaRbqabaGcdaqadaqaaiabdogaJbGaayjkaiaawMcaaaWcbaGaem4AaSgabeqdcqGHris5aaGccaGLOaGaayzkaaaacaGL7bGaayzFaaaaleaacqWGPbqAcqGH9aqpcqaIXaqmaeaacqWGmbata0GaeyyeIuoakiaaxMaacaWLjaWaaeWaaeaacqaIYaGmcqaI4aaoaiaawIcacaGLPaaaaaa@6C8B@

#### Optimal Accuracy Posterior Decoder algorithm

∀k≠B,i=0:AB(0)=0,Ak(0)=−∞∀1≤i≤L:Al(i)=P(yi=cl|x,θ)+max⁡k{Ak(i−1)δ(k,l)}     (29)P(x,πOAPD|θ)=max⁡k{Ak(L)δ(k,E)}
 MathType@MTEF@5@5@+=feaafiart1ev1aaatCvAUfKttLearuWrP9MDH5MBPbIqV92AaeXatLxBI9gBaebbnrfifHhDYfgasaacH8akY=wiFfYdH8Gipec8Eeeu0xXdbba9frFj0=OqFfea0dXdd9vqai=hGuQ8kuc9pgc9s8qqaq=dirpe0xb9q8qiLsFr0=vr0=vr0dc8meaabaqaciaacaGaaeqabaqabeGadaaakqaabeqaaiabgcGiIiabdUgaRjabgcMi5kabdkeacjabcYcaSiabdMgaPjabg2da9iabicdaWiabcQda6iabdgeabnaaBaaaleaacqWGcbGqaeqaaOWaaeWaaeaacqaIWaamaiaawIcacaGLPaaacqGH9aqpcqaIWaamcqGGSaalcqWGbbqqdaWgaaWcbaGaem4AaSgabeaakmaabmaabaGaeGimaadacaGLOaGaayzkaaGaeyypa0JaeyOeI0IaeyOhIukabaGaeyiaIiIaeGymaeJaeyizImQaemyAaKMaeyizImQaemitaWKaeiOoaOJaemyqae0aaSbaaSqaaiabdYgaSbqabaGcdaqadaqaaiabdMgaPbGaayjkaiaawMcaaiabg2da9iabdcfaqnaabmaabaGaemyEaK3aaSbaaSqaaiabdMgaPbqabaGccqGH9aqpcqWGJbWydaahaaWcbeqaaiabdYgaSbaakiabcYha8Hqabiab=Hha4jabcYcaSGGaciab+H7aXbGaayjkaiaawMcaaiabgUcaRmaaxababaGagiyBa0MaeiyyaeMaeiiEaGhaleaacqWGRbWAaeqaaOWaaiWaaeaacqWGbbqqdaWgaaWcbaGaem4AaSgabeaakmaabmaabaGaemyAaKMaeyOeI0IaeGymaedacaGLOaGaayzkaaGae4hTdq2aaeWaaeaacqWGRbWAcqGGSaalcqWGSbaBaiaawIcacaGLPaaaaiaawUhacaGL9baacaWLjaGaaCzcamaabmaabaGaeGOmaiJaeGyoaKdacaGLOaGaayzkaaaabaGaemiuaa1aaeWaaeaacqWF4baEcqGGSaalcqGFapaCdaahaaWcbeqaaiabd+eapjabdgeabjabdcfaqjabdseaebaakiabcYha8jab+H7aXbGaayjkaiaawMcaaiabg2da9maaxababaGagiyBa0MaeiyyaeMaeiiEaGhaleaacqWGRbWAaeqaaOWaaiWaaeaacqWGbbqqdaWgaaWcbaGaem4AaSgabeaakmaabmaabaGaemitaWeacaGLOaGaayzkaaGae4hTdq2aaeWaaeaacqWGRbWAcqGGSaalcqWGfbqraiaawIcacaGLPaaaaiaawUhacaGL9baaaaaa@A481@

As one can observe comparing Equations (25) and (28), the main differences of this algorithm compared to the Posterior-Viterbi is, i) the fact that uses the sums of the posterior label probabilities instead of the posterior probabilities, and ii) that instead of the product, it maximises the sum of these probabilities. Consequently, it is straightforward once again to obtain constraint predictions utilising the prior information:

#### Modified Optimal Accuracy Posterior Decoder algorithm

∀k≠B,i=0:AB(0)=0,Ak(0)=−∞∀1≤i≤L:Al(i)=P(yi=cl|x,ω,θ)+dl(i)max⁡k{Akω(i−1)δ(k,l)}     (30)P(x,ω,πOAPD|θ)=max⁡k{Akω(L)δ(k,E)}
 MathType@MTEF@5@5@+=feaafiart1ev1aaatCvAUfKttLearuWrP9MDH5MBPbIqV92AaeXatLxBI9gBaebbnrfifHhDYfgasaacH8akY=wiFfYdH8Gipec8Eeeu0xXdbba9frFj0=OqFfea0dXdd9vqai=hGuQ8kuc9pgc9s8qqaq=dirpe0xb9q8qiLsFr0=vr0=vr0dc8meaabaqaciaacaGaaeqabaqabeGadaaakqaabeqaaiabgcGiIiabdUgaRjabgcMi5kabdkeacjabcYcaSiabdMgaPjabg2da9iabicdaWiabcQda6iabdgeabnaaBaaaleaacqWGcbGqaeqaaOWaaeWaaeaacqaIWaamaiaawIcacaGLPaaacqGH9aqpcqaIWaamcqGGSaalcqWGbbqqdaWgaaWcbaGaem4AaSgabeaakmaabmaabaGaeGimaadacaGLOaGaayzkaaGaeyypa0JaeyOeI0IaeyOhIukabaGaeyiaIiIaeGymaeJaeyizImQaemyAaKMaeyizImQaemitaWKaeiOoaOJaemyqae0aaSbaaSqaaiabdYgaSbqabaGcdaqadaqaaiabdMgaPbGaayjkaiaawMcaaiabg2da9iabdcfaqnaabmaabaGaemyEaK3aaSbaaSqaaiabdMgaPbqabaGccqGH9aqpcqWGJbWydaahaaWcbeqaaiabdYgaSbaakiabcYha8Hqabiab=Hha4jabcYcaSGGabiab+L8a3jabcYcaSGGaciab9H7aXbGaayjkaiaawMcaaiabgUcaRiabdsgaKnaaBaaaleaacqWGSbaBaeqaaOWaaeWaaeaacqWGPbqAaiaawIcacaGLPaaadaWfqaqaaiGbc2gaTjabcggaHjabcIha4bWcbaGaem4AaSgabeaakmaacmaabaGaemyqae0aa0baaSqaaiabdUgaRbqaaiab9L8a3baakmaabmaabaGaemyAaKMaeyOeI0IaeGymaedacaGLOaGaayzkaaGae0hTdq2aaeWaaeaacqWGRbWAcqGGSaalcqWGSbaBaiaawIcacaGLPaaaaiaawUhacaGL9baacaWLjaGaaCzcamaabmaabaGaeG4mamJaeGimaadacaGLOaGaayzkaaaabaGaemiuaa1aaeWaaeaacqWF4baEcqGGSaalcqGFjpWDcqGGSaalcqqFapaCdaahaaWcbeqaaiabd+eapjabdgeabjabdcfaqjabdseaebaakiabcYha8jab9H7aXbGaayjkaiaawMcaaiabg2da9maaxababaGagiyBa0MaeiyyaeMaeiiEaGhaleaacqWGRbWAaeqaaOWaaiWaaeaacqWGbbqqdaqhaaWcbaGaem4AaSgabaGae0xYdChaaOWaaeWaaeaacqWGmbataiaawIcacaGLPaaacqqF0oazdaqadaqaaiabdUgaRjabcYcaSiabdweafbGaayjkaiaawMcaaaGaay5Eaiaaw2haaaaaaa@B31F@

### Technical comments on the implementation and future improvements

There are various ways, with which the delta function described in Equation (8) can be implemented. The most obvious is to introduce a new matrix, similar to those used in all the algorithms (forward, backward etc), in which we store the values of the function. Another way (which in many cases may be more preferable) is to perform the appropriate tests for the agreement of labels with states along the sequence, each time we visit a particular state. Furthermore, with the general notation used in Equation (8), the algorithms described here may also be used in situations where the label probability is not a delta function but instead we allow one state to match more than one labels, requiring thus a full probability distribution of the form:

*λ*_*k*_(c) = *P*(*y*_*i *_= *c*|*π*_*i *_= *k*)

In such situations, all the algorithms described here remain unchanged.

Another useful note arises from the observation that in some situations the localisation of a particular residue or segment cannot be easily assigned to one of the model's labels using certain experimental techniques. For instance, using fusions of beta-lactamase (BlaM) in a potential loop and observing negative activity of the fused domain, we may interpret the results as indication for the localisation into the cytoplasm. Unfortunately, the same results could be observed if the particular residue was localised in a transmembrane segment. Similar interpretations may also occur for other reporter fusions (GFP, LacZ etc). In such situations, the above algorithms may also be extended to incorporate this information, by simply allowing the observation to consist of a set of possible labels for each position along the sequence. For instance, instead of stating that the localisation of residue *i *is *cytoplasmic *(*I*):

*ω*_*i *_= *I*

we may state that

*ω*_*i *_= {*M,I*} = *O*^*C*^

meaning that the localisation is *not extracellular *(*O*^*C*^). Modifying now properly the delta function in Equation (8), all the algorithms presented above remain unchanged. We also have to note that the above-mentioned algorithms remain unchanged in cases where the HMM uses continuous instead of discrete emission probabilities, indicating the general applicability of our approach. Finally, we have to mention also a further addition to the algorithms developed here. In particular, if we replace the delta function in Equation (8) with a full probability distribution, all the algorithms presented above remain unchanged and the probabilistic interpretation of the model is retained. This situation may arise in cases where we have multiple sources of information available, and we have reasons for assigning different weights to each one (for instance if we have reasons to believe that one method is more reliable than another). Although this might be a useful strategy, especially in cases of conflicting or ambiguous experiments, we have not tried to investigate this further.

### The model architecture and training procedure

**Figure 4:**
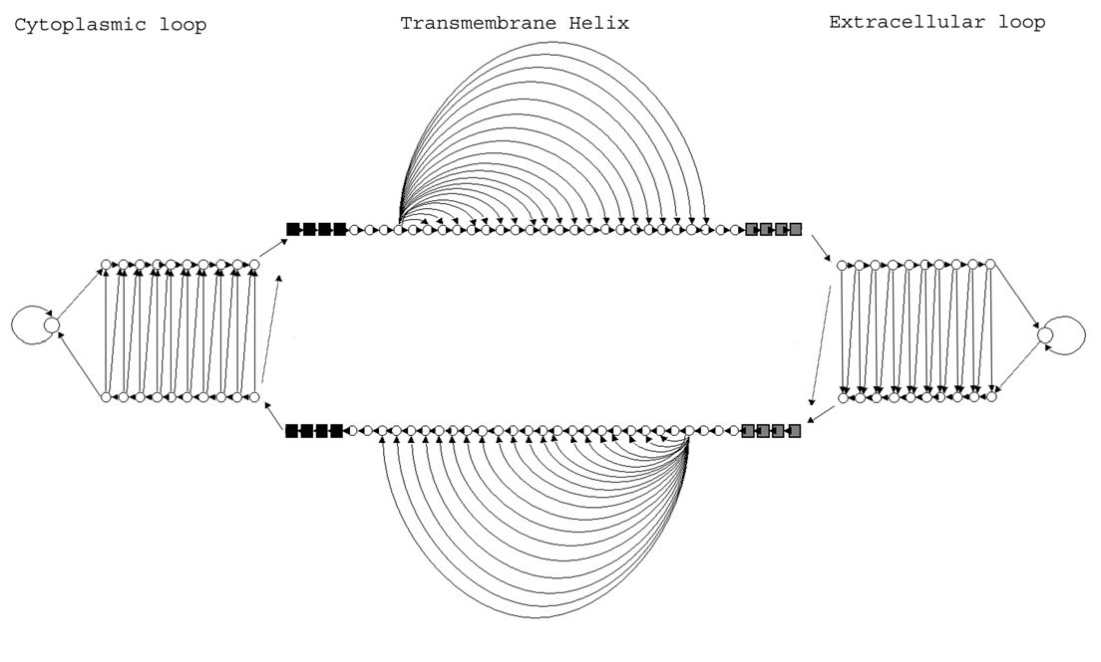
**A schematic representation of the model's architecture**. The model consists of three sub-models denoted by the labels: Cytoplasmic loop, Transmembrane Helix and Extracellular loop. Within each sub-model, states with the same shape, size and colour are sharing the same emission probabilities (parameter tying). Allowed transitions are indicated with arrows.

In order to show the effectiveness of the modified algorithms that we described so far, we developed a method to predict the membrane-spanning segments of alpha-helical membrane proteins. The model that we used is cyclic, consisting of 114 states, including begin (B) and end (E) states, (Figure 4), and is conceptually similar to other models described earlier for the same task [9,11,54], even though somewhat simpler. The architecture has been chosen so that it could fit as much as possible to the limitations imposed by the known structures and the prior biological knowledge. The model consists of three "sub-models" corresponding to the three desired labels to predict, the TM (transmembrane) helix sub-model and the inner and outer loops sub-models respectively. The TM helix model incorporates states to model the architecture of the transmembrane helices. Thus, there are states that correspond to the core of the helix and the cap located at the lipid bilayer interface. All states are connected with the appropriate transition probabilities in order to be consistent with the known structures, that is, to ensure appropriate length distribution. The inner and outer loops are modelled with a "ladder" architecture, at the top each is a self transitioning state corresponding to residues too distant from the membrane; these cannot be modelled as loops, hence that state is named "globular".

For training the model we used the Conditional Maximum Likelihood (CML) criterion for labelled data [32,33], with the modified algorithms for faster and robust training described in [55]. The training procedure consisted of three steps. Briefly, a model was initially estimated using the Baum-Welch algorithm for labelled sequences [32]. Afterwards, the labels of the sequences were deleted in a region flanking three residues in each direction of the end of a membrane-spanning helix, and predictions were performed using the modified Viterbi algorithm presented above, and the model that was estimated from step 1. The final model was estimated, using the labels derived from step 2, with the modified gradient-descent method for CML training [55]. Finally the decoding is performed with the Optimal Accuracy Posterior Decoder, as described in [36].

### Datasets

The training set that we used contains 72 membrane proteins with three dimensional structures determined at atomic resolution and most of these structures are deposited in the Protein Data Bank (PDB) [56]. The dataset is the one used in the work of [38], except from one protein (1PGE:A) that was not considered to be a typical membrane protein (possesses transmembrane helices shorter than 10 amino-acids). In all cases the sequence used, was that obtained from Uniprot [57], after the removal of any signal peptide, that could bias the training and testing procedure [58]. The training dataset consists of 39 single spanning and 33 multi-spanning membrane proteins, of which 42 are of prokaryotic (including viruses) and 30 of eukaryotic origin. Thus, the dataset is considered representative and is of similar composition compared to the training set used for most of the other top-scoring HMM predictors [9,11,13,29,39]. However, it is significantly smaller consisting of only 72 proteins, in contrast to the other datasets used that consisted of nearly more than 150 transmembrane proteins. Only Zhou and Zhou [38], which used effectively the same set, and Martelli et al [39], which compiled a dataset of 59 transmembrane proteins, used sets of comparable size. The striking feature of the particular dataset is the fact that consists only of proteins with crystallographically solved structure. Although it is common in the relevant publications to use datasets of mixed resolution, i.e. proteins with three-dimensional structure mixed with proteins with topology determined by low resolution biochemical experiments [9,11,29], the results of Martelli et al [39], clearly showed that the low resolution sets are less reliable and they should not be used either as training or even as test sets.

In order to compare the accuracy of the developed method and facilitate the unbiased comparison against the other established methods, we compiled a dataset of transmembrane proteins whose three-dimensional structures are available and deposited in PDB. We chose proteins, which do not show significant similarity (<30% identities in length of more than 80 residues), to any of the proteins used for training either by the current method (and by UMDHMM^TMHP^), by HMMTOP, or by TMHMM. This dataset consists of 26 proteins (Table 3), with numbers of transmembrane segments ranging from 1–14, and is considered to be an as representative as possible (although small) set, on which the performance of the various predictors could be evaluated in an unbiased manner.

As a further blind test we also used a set of 34 *E.coli *inner membrane proteins [26] and a set of 39 cytoplasmic membrane proteins of *S. cerevisiae *[27], with experimentally verified localization of the C-terminus. This set contains proteins of which the topology are not known, with the exception of the C-terminal part, but the majority of the web-predictors evaluated in the respective study agree for the number of their transmembrane helices. For 3 proteins of the *E. coli *and 2 of the *S. cerevisiae *set, the localisation of the C-terminus could not be obtained, thus, for these proteins only the number of predicted transmembrane segments are considered. The primary intention of using this set was to evaluate the accuracy of the various predictors in determining only partially the topology, as well as in order to show that using the prior knowledge about the topology the prediction accuracy increases.

For evaluating the ability of the methods to correctly discriminate transmembrane proteins from globular ones, i.e. to evaluate the rate of false positive predictions, we used an additional non-redundant dataset of 645 globular proteins with crystallographically solved structures, that was initially used by the developers of TMHMM [9].

In all of the comparisons performed, the evaluation of the statistical significance of the results was performed using the Kruskal-Wallis non-parametric test for the equality of distributions between several populations. This test is the non-parametric analogue of the one-way Analysis of Variance (ANOVA), and was chosen as it best fits the nature of the data we compare (non-normally distributed) as well as the relative small sample size. Statistical significance was declared for p-values < 0.05.

## Authors' contributions

PB formulated the algorithms, drafted the manuscript, performed the analysis, and participated in the implementation. TL participated in the analysis, and implemented most of the algorithms as well as the web-interface. SH supervised the whole project, and participated in drafting the manuscript. All authors have read and accepted the final manuscript.

## Supplementary Material

Additional File 1 containing the detailed results obtained using the available predictors, on the 2 datasets of proteins with experimentally verified localisation of the C-terminus (supplement.xls). Click here for file

## Figures and Tables

**Table 1:** Results obtained from the various predictors, on a dataset of 72 transmembrane proteins [38]. Results obtained when the methods were not trained and tested on the same dataset, however some of the proteins in the dataset were present in the datasets used for training the other methods. The results of HMM-TM were obtained through a nine-fold cross validation procedure. The methods that allow the incorporation of experimental information are listed separately. The results of UMDHMM^TMHP ^could not be obtained by cross-validation (since it was trained on the same dataset), and thus are listed separately in the text

**Method**	**Q**	**C**	**SOV**	**Correctly predicted TM segments (%)**	**Correctly predicted Topologies (%)**
***Methods that allow the incorporation of experimental information***					
**HMM-TM (cross-validation)**	**0.903**	**0.762**	**0.939**	**59/72 (81.9%)**	**55/72 (76.4%)**
TMHMM	0.902	0.762	0.931	58/72 (80.6%)	49/72 (68.1%)
HMMTOP	0.890	0.735	0.932	58/72 (80.6%)	49/72 (68.1%)
Phobius †	0.911	0.785	0.954	65/72 (90.3%)	52/72 (72.2%)
***Methods that do not allow the incorporation of experimental information***					
MEMSAT	0.905	0.767	0.954	63/72 (87.5%)	48/72 (66.7%)
S-TMHMM †	0.897	0.747	0.925	59/72 (81.9%)	52/72 (72.2%)
PRO-TMHMM* †	0.910	0.779	0.945	65/72 (90.3%)	63/72 (87.5%)
PRODIV-TMHMM* †	0.914	0.794	0.970	67/72 (93.1%)	64/72 (87.5%)

* The methods using evolutionary information are denoted with an asterisk.

† These predictors were trained on sets containing sequences similar to the ones included in the training set we used here

**Table 2:** Results of the independent test on a dataset of 26 transmembrane proteins with known three-dimensional structures. The proteins were chosen not to have significant sequence identity (<30%) with the proteins used to train the methods: HMM-TM, UMDHMM^TMHP^, TMHMM and HMMTOP. The methods that allow the incorporation of experimental information are listed separately

**Method**	**Q**	**C**	**SOV**	**Correctly predicted TM segments (%)**	**Correctly predicted Topologies (%)**
**Methods that allow the incorporation of experimental information**					
**HMM-TM**	**0.899**	**0.780**	**0.942**	**21/26 (80.77%)**	**21/26 (80.77%)**
TMHMM	0.899	0.782	0.956	19/26 (73.08%)	17/26 (65.38%)
HMMTOP	0.881	0.744	0.925	19/26 (73.08%)	18/26 (69.23%)
Phobius †	0.894	0.773	0.907	15/26 (57.69%)	13/26 (50%)
**Methods that do not allow the incorporation of experimental information**					
MEMSAT	0.890	0.762	0.928	16/26 (61.54%)	13/26 (50%)
UMDHMM^TMHP^	0.896	0.777	0.947	23/26 (88.46%)	22/26 (84.61%)
S-TMHMM †	0.899	0.781	0.957	21/26 (80.77%)	20/26 (76.92%)
PRO-TMHMM*†	0.870	0.718	0.916	16/26 (61.54%)	15/26 (57.69%)
PRODIV-TMHMM*†	0.897	0.778	0.946	19/26 (73.08%)	19/26 (73.08%)

* The methods using evolutionary information are denoted with an asterisk.

† These predictors were trained on sets containing sequences similar to the ones included in the test set.

**Table 3:** The independent test set of 26 transmembrane proteins with known three-dimensional structures. We list the PDB code, the name of the protein and the number of the transmembrane segments

**PDB code**	**Name**	**Number of transmembrane segments**
1RC2:B	AQUAPORIN Z	6
1J4N:A	AQUAPORIN 1	6
1IWG:A	MULTIDRUG EFFLUX TRANSPORTER ACRB	12
1NEK:C	SUCCINATE DEHYDROGENASE CYTOCHROME B-556 SUBUNIT	3
1NEK:D	SUCCINATE_DEHYDROGENASE HYDROPHOBIC MEMBRANE ANCHOR PROTEIN	3
1Q16:C	RESPIRATORY NITRATE REDUCTASE 1 GAMMA CHAIN	5
1RH5:B	PREPROTEIN TRANSLOCASE SECE SUBUNIT	1
1RH5:C	PREPROTEIN TRANSLOCASE SECBETA SUBUNIT	1
1IZL:A	PHOTOSYSTEM II SUBUNIT PSBA	5
1IZL:C	PHOTOSYSTEM II SUBUNIT PSBC	5
1YCE	ROTOR OF F-TYPE NA+-ATPASE	2
2BL2	ROTOR OF V-TYPE NA+-ATPASE	4
1XQF:A	PROBABLE AMMONIUM TRANSPORTER	11
1KPL:A	H+/CL- EXCHANGE TRANSPORTER	14
1S5L:B	PHOTOSYSTEM II CORE LIGHT HARVESTING PROTEIN	6
1S5L:C	PHOTOSYSTEM II CP43 PROTEIN	6
1S5L:D	PHOTOSYSTEM II REACTION CENTER D2 PROTEIN	5
1S5L:E	CYTOCHROME B559 ALPHA SUBUNIT	1
1FX8:A	GLPF GLYCEROL FACILITATOR CHANNEL	6
1LNQ:A	MTHK POTTASIUM CHANNEL, CA-GATED	2
1MXM:A	MECHANOSENSITIVE CHANNEL PROTEIN	3
1Q90:M	CYTOCHROME B6F COMPLEX SUBUNIT PETM	1
1VF5:C	CYTOCHROME F	1
1K4C:C	POTASSIUM CHANNEL KCSA	2
1ZCD:A	NA(+)/H(+) ANTIPORTER 1	12
2A79:B	POTASSIUM VOLTAGE-GATED CHANNEL SUBFAMILY A MEMBER 2	6

## References

[B1] Rabiner LR (1989). A tutorial on hidden Markov models and selected applications in speech recognition.. Proc IEEE.

[B2] Durbin R, Eddy SR, Krogh A, Mithison G (1998). Biological sequence analysis, probabilistic models of proteins and nucleic acids.

[B3] Krogh A, Mian IS, Haussler D (1994). A hidden Markov model that finds genes in E. coli DNA. Nucleic Acids Res.

[B4] Eddy SR (1995). Multiple alignment using hidden Markov models. Proc Int Conf Intell Syst Mol Biol.

[B5] Eddy SR (1998). Profile hidden Markov models. Bioinformatics.

[B6] Juncker AS, Willenbrock H, von Heijne G, Brunak S, Nielsen H, Krogh A (2003). Prediction of lipoprotein signal peptides in Gram-negative bacteria. Protein Sci.

[B7] Nielsen H, Krogh A (1998). Prediction of signal peptides and signal anchors by a hidden Markov model. Proc Int Conf Intell Syst Mol Biol.

[B8] Asai K, Hayamizu S, Handa K (1993). Prediction of protein secondary structure by the hidden Markov model. Comput Appl Biosci.

[B9] Krogh A, Larsson B, von Heijne G, Sonnhammer EL (2001). Predicting transmembrane protein topology with a hidden Markov model: application to complete genomes. J Mol Biol.

[B10] Bagos PG, Liakopoulos TD, Spyropoulos IC, Hamodrakas SJ (2004). A Hidden Markov Model method, capable of predicting and discriminating beta-barrel outer membrane proteins. BMC Bioinformatics.

[B11] Kall L, Krogh A, Sonnhammer EL (2004). A combined transmembrane topology and signal peptide prediction method. J Mol Biol.

[B12] Moller S, Croning MD, Apweiler R (2001). Evaluation of methods for the prediction of membrane spanning regions. Bioinformatics.

[B13] Viklund H, Elofsson A (2004). Best alpha-helical transmembrane protein topology predictions are achieved using hidden Markov models and evolutionary information. Protein Sci.

[B14] Bagos PG, Liakopoulos TD, Hamodrakas SJ (2005). Evaluation of methods for predicting the topology of ß-barrel outer membrane proteins and a consensus prediction method. BMC Bioinformatics.

[B15] Traxler B, Boyd D, Beckwith J (1993). The topological analysis of integral cytoplasmic membrane proteins. J Membr Biol.

[B16] van Geest M, Lolkema JS (2000). Membrane topology and insertion of membrane proteins: search for topogenic signals. Microbiol Mol Biol Rev.

[B17] Bennett KL, Matthiesen T, Roepstorff P (2000). Probing protein surface topology by chemical surface labeling, crosslinking, and mass spectrometry. Methods Mol Biol.

[B18] Jarvik JW, Telmer CA (1998). Epitope tagging. Annu Rev Genet.

[B19] Conti-Fine BM, Lei S, McLane KE (1996). Antibodies as tools to study the structure of membrane proteins: the case of the nicotinic acetylcholine receptor. Annu Rev Biophys Biomol Struct.

[B20] Loo TW, Clarke DM (1999). Determining the structure and mechanism of the human multidrug resistance P-glycoprotein using cysteine-scanning mutagenesis and thiol-modification techniques. Biochim Biophys Acta.

[B21] Manoil C (1991). Analysis of membrane protein topology using alkaline phosphatase and beta-galactosidase gene fusions. Methods Cell Biol.

[B22] Broome-Smith JK, Tadayyon M, Zhang Y (1990). Beta-lactamase as a probe of membrane protein assembly and protein export. Mol Microbiol.

[B23] Ki JJ, Kawarasaki Y, Gam J, Harvey BR, Iverson BL, Georgiou G (2004). A periplasmic fluorescent reporter protein and its application in high-throughput membrane protein topology analysis. J Mol Biol.

[B24] Melen K, Krogh A, von Heijne G (2003). Reliability measures for membrane protein topology prediction algorithms. J Mol Biol.

[B25] Drew D, Sjostrand D, Nilsson J, Urbig T, Chin CN, de Gier JW, von Heijne G (2002). Rapid topology mapping of Escherichia coli inner-membrane proteins by prediction and PhoA/GFP fusion analysis. Proc Natl Acad Sci U S A.

[B26] Rapp M, Drew D, Daley DO, Nilsson J, Carvalho T, Melen K, De Gier JW, Von Heijne G (2004). Experimentally based topology models for E. coli inner membrane proteins. Protein Sci.

[B27] Kim H, Melen K, von Heijne G (2003). Topology models for 37 Saccharomyces cerevisiae membrane proteins based on C-terminal reporter fusions and predictions. J Biol Chem.

[B28] Daley DO, Rapp M, Granseth E, Melen K, Drew D, von Heijne G (2005). Global topology analysis of the Escherichia coli inner membrane proteome. Science.

[B29] Tusnady GE, Simon I (2001). The HMMTOP transmembrane topology prediction server. Bioinformatics.

[B30] TMHMMfix http://www.sbc.su.se/TMHMMfix/.

[B31] Bernsel A, Von Heijne G (2005). Improved membrane protein topology prediction by domain assignments. Protein Sci.

[B32] Krogh A (1994). Hidden Markov models for labelled sequences.. Proceedings of the12th IAPR International Conference on Pattern Recognition.

[B33] Krogh A (1997). Two methods for improving performance of an HMM and their application for gene finding. Proc Int Conf Intell Syst Mol Biol.

[B34] Fariselli P, Finelli M, Marchignoli D, Martelli PL, Rossi I, Casadio R (2003). MaxSubSeq: an algorithm for segment-length optimization. The case study of the transmembrane spanning segments. Bioinformatics.

[B35] Fariselli P, Martelli PL, Casadio R (2005). A new decoding algorithm for hidden Markov models improves the prediction of the topology of all-beta membrane proteins. BMC Bioinformatics.

[B36] Kall L, Krogh A, Sonnhammer EL (2005). An HMM posterior decoder for sequence feature prediction that includes homology information. Bioinformatics.

[B37] Jones DT, Taylor WR, Thornton JM (1994). A model recognition approach to the prediction of all-helical membrane protein structure and topology. Biochemistry.

[B38] Zhou H, Zhou Y (2003). Predicting the topology of transmembrane helical proteins using mean burial propensity and a hidden-Markov-model-based method. Protein Sci.

[B39] Martelli PL, Fariselli P, Casadio R (2003). An ENSEMBLE machine learning approach for the prediction of all-alpha membrane proteins. Bioinformatics.

[B40] Rost B, Casadio R, Fariselli P (1996). Refining neural network predictions for helical transmembrane proteins by dynamic programming. Proc Int Conf Intell Syst Mol Biol.

[B41] Rost B, Fariselli P, Casadio R (1996). Topology prediction for helical transmembrane proteins at 86% accuracy. Protein Sci.

[B42] Claros MG, von Heijne G (1994). TopPred II: an improved software for membrane protein structure predictions. Comput Appl Biosci.

[B43] Baldi P, Brunak S, Chauvin Y, Andersen CA, Nielsen H (2000). Assessing the accuracy of prediction algorithms for classification: an overview. Bioinformatics.

[B44] Zemla A, Venclovas C, Fidelis K, Rost B (1999). A modified definition of Sov, a segment-based measure for protein secondary structure prediction assessment. Proteins.

[B45] Murakami S, Nakashima R, Yamashita E, Yamaguchi A (2002). Crystal structure of bacterial multidrug efflux transporter AcrB. Nature.

[B46] Fujihira E, Tamura N, Yamaguchi A (2002). Membrane topology of a multidrug efflux transporter, AcrB, in Escherichia coli. J Biochem (Tokyo).

[B47] Promponas VJ, Palaios GA, Pasquier CM, Hamodrakas JS, Hamodrakas SJ (1999). CoPreTHi: a Web tool which combines transmembrane protein segment prediction methods. In Silico Biol.

[B48] Nilsson J, Persson B, von Heijne G (2000). Consensus predictions of membrane protein topology. FEBS Lett.

[B49] Arai M, Mitsuke H, Ikeda M, Xia JX, Kikuchi T, Satake M, Shimizu T (2004). ConPred II: a consensus prediction method for obtaining transmembrane topology models with high reliability. Nucleic Acids Res.

[B50] Cuff JA, Clamp ME, Siddiqui AS, Finlay M, Barton GJ (1998). JPred: a consensus secondary structure prediction server. Bioinformatics.

[B51] Zheng WJ, Spassov VZ, Yan L, Flook PK, Szalma S (2004). A hidden Markov model with molecular mechanics energy-scoring function for transmembrane helix prediction. Comput Biol Chem.

[B52] Schwartz R, Chow YL (1990). The N-Best Algorithm: An Efficient and Exact Procedure for Finding the N Most Likely Sentence Hypotheses.. Proc IEEE Int Conf Acoust, Speech, Sig Proc.

[B53] Bagos PG, Liakopoulos TD, Spyropoulos IC, Hamodrakas SJ (2004). PRED-TMBB: a web server for predicting the topology of beta-barrel outer membrane proteins. Nucleic Acids Res.

[B54] Tusnady GE, Simon I (1998). Principles governing amino acid composition of integral membrane proteins: application to topology prediction. J Mol Biol.

[B55] Bagos PG, Liakopoulos TD, Hamodrakas SJ, Paliouras G, Sakakibara Y (2004). Faster Gradient  Descent Conditional Maximum Likelihood  Training of Hidden Markov Models, Using Individual Learning Rate Adaptation: Athens..

[B56] Berman HM, Battistuz T, Bhat TN, Bluhm WF, Bourne PE, Burkhardt K, Feng Z, Gilliland GL, Iype L, Jain S, Fagan P, Marvin J, Padilla D, Ravichandran V, Schneider B, Thanki N, Weissig H, Westbrook JD, Zardecki C (2002). The Protein Data Bank. Acta Crystallogr D Biol Crystallogr.

[B57] Bairoch A, Apweiler R, Wu CH, Barker WC, Boeckmann B, Ferro S, Gasteiger E, Huang H, Lopez R, Magrane M, Martin MJ, Natale DA, O'Donovan C, Redaschi N, Yeh LS (2005). The Universal Protein Resource (UniProt). Nucleic Acids Res.

[B58] Lao DM, Arai M, Ikeda M, Shimizu T (2002). The presence of signal peptide significantly affects transmembrane topology prediction. Bioinformatics.

